# Supplementary material for: What is usual care for teenagers expecting their first child in England? A process evaluation using key informant mapping and participant survey as part of the Building Blocks randomised controlled trial of specialist home visiting
Source: BMJ Open. 2018 May 5;8(5):e020152. doi: 10.1136/bmjopen-2017-020152 (PMC5942429; doi:10.1136/bmjopen-2017-020152)
Supplement: Supplementary data [file bmjopen-2017-020152supp001.pdf]

Appendix 1

Example of service mapping content / user instructions from ‘Education’ worksheet

Please describe services available for teenage, first-time mothers in your area	Name of service (If applicable)	Description of service / Care	Are there any limits on the availability of this service? E.g., Number of women that can be offered the service
Is there a Mother and Baby hostel in your area?	Mother and baby hostels	Dedicated hostels for homeless women who are pregnant or have a new baby. Additional support both from trained staff	Some will take young women in the early stages of pregnancy (up to 6 months approx). Some will not house pregnant women, or young babies
What temporary accommodation can be offered?	Hostels	Temporary accommodation with varying degrees of support, usually containing some shared facilities	Most require assessment of eligibility and suitability. Not usually suitable for those under 18, or youths with offending behaviour.
Temporary accommodation (for the temporary homeless)?	Emergency hostels and night shelters (or bed and breakfasts if nothing else is available - usu max of 6 weeks)	Offer somewhere to sleep, food, warmth and hygiene. Residents are normally asked to pay a small additional contribution for their meals.	Usually a direct access/first-come-first served basis. Night shelters are usually free. Most hostels charge.
Charity involvement?	LIFE Housing	Provides a support service (General Support, Individual Support Plans and the LIFE Skills Programme) and community outreach schemes	(Not specified)
Foyers	Foyers	Integration of accommodation and support services: training in basic/independent living skills, inc. ongoing support when the young person has left the Foyer	Most foyers have a waiting list. Some foyers only accept referrals from local councils
Women's refuges	Women's refuges	A refuge is a safe house where women and children who are experiencing domestic violence can stay free from abuse	Refuges are highly unlikely to accept women from their immediate local area. Some are for women w/ part' ethnic/cultural backgrounds
Housing Associations / RSLs (Registered Social Landlords)	Housing associations / RSLs (Registered Social Landlords)	Provide homes for people on low incomes. Some housing associations specialise in accommodation for particular groups of people, such as younger people	Long waiting list. Chance of place/waiting time depends on personal circumstances (e.g. children), and other factors
Supported lodgings schemes	Supported lodgings	Individuals in the community offer a room in their home with varying degrees of support. A safe and supportive environment for young people	Suitable for youths: leaving care, deemed vulnerable and in need, requiring temp acc, or with no statutory entitlement to housing

Supported housing	Self-contained, cluster and shared arrangements with varying degrees of support	Supported housing will usually provide housing-related support to help a young person prepare for independent living	Many schemes accessible via social services' leaving care arrangements, and via housing departments for young homeless people
Mainstream housing	Independent accommodation	Independent accommodation. Housing & social services may have arrangements w/ private landlords to provide accommodation for vulnerable youths	Housing benefit restrictions apply to under 25s living in the private sector and applicants are limited to the single-room rent housing benefit level
Nightstop schemes	Emergency accommodation (Depaul Nightstop UK)	Nightstop schemes provide emergency accommodation for young homeless people aged 16–25 in the homes of a network of volunteer hosts	Referrals to service after risk assessments by a recognised agency; such as social services, Connexions, police, housing depts
Supported housing	Supported housing schemes	Housing schemes offering accommodation linked with on-site or outreach support from dedicated staff (practical and emotional help)	Schemes vary in size
Custodial institution	Secure Children's Homes	Concentrate on physical, emotional, behavioural needs, & aim to give youths individually tailored support to resolve the issues that led them to commit an offence	These are relatively small institutions, with between 6 and 40 beds and a high staff to young person ratio
Floating support	Floating support services	To sustain a tenancy through the dev' of independent living skills. Provides general, non-specialist support with daily living skills, practical tasks or emotional support which promotes or maintains a person's ability to live in their own home	Reviews of the progress and support plan ensure that the services adjust appropriately to changing needs. An exit strategy determines when support is withdrawn. If a person needs support later, it can return to them
