# Supplementary material for: What is usual care for teenagers expecting their first child in England? A process evaluation using key informant mapping and participant survey as part of the Building Blocks randomised controlled trial of specialist home visiting
Source: BMJ Open. 2018 May 5;8(5):e020152. doi: 10.1136/bmjopen-2017-020152 (PMC5942429; doi:10.1136/bmjopen-2017-020152)
Supplement: Author's manuscript [file bmjopen-2017-020152.draft_revisions.pdf]

BMJ Open is committed to open peer review. As part of this commitment we make the peer review history of every article we publish publicly available.

When an article is published we post the peer reviewers' comments and the authors' responses online. We also post the versions of the paper that were used during peer review. These are the versions that the peer review comments apply to.

The versions of the paper that follow are the versions that were submitted during the peer review process. They are not the versions of record or the final published versions. They should not be cited or distributed as the published version of this manuscript.

BMJ Open is an open access journal and the full, final, typeset and author-corrected version of record of the manuscript is available on our site with no access controls, subscription charges or pay-per-view fees (http://bmjopen.bmj.com).

If you have any questions on BMJ Open's open peer review process please email info.bmjopen@bmj.com

BMJ Open

Understanding and measuring usual care in a trial of a complex home visiting intervention: key informant mapping and participant survey within a randomised controlled trial

Journal:	*BMJ Open*
Manuscript ID	bmjopen-2017-020152
Article Type:	Research
Date Submitted by the Author:	27-Oct-2017
Complete List of Authors:	Robling, Michael; Cardiff University, Centre for Trials Research<br>Cannings John, Rebeca; Cardiff University, Centre for Trials Research<br>Channon, Sue; Cardiff University, Centre for Trials Research<br>Hood, Kerenza; Cardiff University, South East Wales Trials Unit, Centre for Trials Research<br>Moody, Gwenllian; Cardiff University, Centre for Trials Research<br>Poole, Ria; Cardiff University, Medicine<br>Sanders, Julia; School of Healthcare Sciences, Room 12:15, Eastgate House, 35-43 Newport Road
Keywords:	Organisation of health services < HEALTH SERVICES ADMINISTRATION & MANAGEMENT, PAEDIATRICS, Child protection < PAEDIATRICS, PUBLIC HEALTH

SCHOLARONE™ Manuscripts

c:\programdata\activepdf\dc_ent\tmp\6c500b\27691771_file000002_639940258.docx

Title: Understanding and measuring usual care in a trial of a complex home visiting intervention: key informant mapping and participant survey within a randomised controlled trial

Corresponding author:

Prof Mike Robling
Centre for Trials Research, Cardiff University
Neuadd Meirionnydd, Heath Park, Cardiff. CF14 4YS
roblingmr@cardiff.ac.uk
+44 (0)29 2068 7177

Authors:

- Mike Robling, Centre for Trials Research, Cardiff University, Cardiff, Wales, UK
- Rebecca Cannings-John, Centre for Trials Research, Cardiff University, Cardiff, Wales, UK
- Sue Channon, Centre for Trials Research, Cardiff University, Cardiff, Wales, UK
- Kerry Hood, Centre for Trials Research, Cardiff University, Cardiff, Wales, UK
- Gwen Moody, Centre for Trials Research, Cardiff University, Cardiff, Wales, UK
- Ria Poole, School of Medicine, Cardiff University, Cardiff, Wales, UK
- Julia Sanders, School of Healthcare Sciences, Cardiff University, Cardiff, Wales, UK

Key words: Public health, child health, nursing, randomised trial

Current abstract word count: 250
Current manuscript word count: 3000
Reference count: 17

For peer review only

c:\programdata\activepdf\dc_ent\tmp\6c500b\27691771_file000002_639940258.docx

Title: Understanding and measuring usual care in a trial of a complex home visiting intervention: key informant mapping and participant survey within a randomised controlled trial

Abstract

Objectives
We compared the US-derived Family Nurse Partnership (FNP) home visiting programme when added to usually provided health and social care in England for teenage first-time mothers, to usual care alone. We aimed to: establish the nature of usual care to clarify what FNP was being added to, measure service usage and assess performance bias in provision of core usual care.

Design
Process evaluation integrated into a trial. Local service professionals completed a survey mapping local health and social care services in seven domains. This focused on services available to young women, especially those relevant to pregnant teenagers. Descriptive data were assessed thematically to establish the range of services. A second round of quantitative data collection with FNP supervisors enumerated service provision by site. Services identified were included in participant trial follow-up interviews to quantify usage.

Setting
Eighteen trial sites in England comprising partnerships of healthcare and local authority organisations.

Outcomes
Descriptive framework of services. Rates of service usage reported by trial participants.

Results
161 separate services were identified, with multiple service models in each domain, broadly categorised as universal or specialist (eg for teenage mothers). FNP supervisors identified 30-63 universal services per site and 22-67 specialist services. Use of core services of maternity care and health visiting show broad equivalence for the former by trial arm and only small differences for the latter. Participants accessed a wide range of services.

Conclusions
A large number of universal and specialist services, across several domains were available and accessed by teenage mothers, potentially limiting the incremental benefit achievable via an enhanced supportive home visiting service. Variation in health visitor support may reflect expected progressive universal support with little evidence of compensatory practice due to FNP's presence. Measuring usual care in complex public health settings is challenging and essential.

For peer review only - http://bmjopen.bmj.com/site/about/guidelines.xhtml

c:\programdata\activepdf\dc_ent\tmp\6c500b\27691771_file000002_639940258.docx

Strengths and limitations of this study

- Local stakeholders with existing knowledge about a range of relevant services have enabled the development of a rich picture of what may be usually available care for teenagers expecting their first child.
- Mapping these services provides a detailed understanding of what the community based control condition in our trial comprised, something which is infrequently available in many trials of home visiting
- Changes over time, and within and between site differences in how services are configured, perceived and understood means that a statement about all locally relevant services is unlikely to be definitive.
- Although we have an understanding about how services were similarly or differently accessed by intervention and control participants in the trial, the intensity and duration of individual sessions for non-FNP services is not known.
- Some bias is likely despite relatively high trial follow-up rates, although other analyses show only minimal differences between those recruited and followed-up.

For peer review only

c:\programdata\activepdf\dc_ent\tmp\6c500b\27691771_file000002_639940258.docx

INTRODUCTION

Individual, social, and economic circumstances faced by teenage mothers can challenge a successful start for their children. Responding in 2006, the Government in England adopted a preventative US-derived programme of nurse-led intensive home-visiting, the Family Nurse Partnership (FNP). Specially trained family nurses support first-time mothers through up to 64 home visits starting in early pregnancy and until the child reaches their second birthday. In three US trials, the programme has been evaluated with differing socio-demographic populations, justifying initial testing in a UK context.[1-3]

Following an implementation evaluation, 18 English sites participated in the Building Blocks trial (ISRCTN23019866) of the programme's effectiveness recruiting 1645 teenagers expecting their first child.[4-8] Women were recruited before 24 weeks gestation, lived within geographical areas served by the FNP team and spoke at least conversational English. Assessing over 60 short-term outcomes (to 24 months post-partum) in domain areas of pregnancy and birth, child development, and maternal life-course, four primary outcomes of programme and policy interest were prioritised.

We compared FNP when added to usually provided health and social care to usual care alone. In the absence of comprehensive public healthcare in the US, across all three previous evaluations the counterfactual was reported as obstetric office-based antenatal care, paediatric developmental screening, referral at specified time points and free transport to office-based consultations. Elevating the control condition to just more than simply no care, the augmented control condition was not further described. Given the provision of free universal health services in the UK, the ethical trial comparator was an active control condition. However, it was expected that what would be available to young families may be complex and vary by site and over time.

We aimed to map and quantify usually provided care and so clarify the trial's control condition, the service context into which FNP was introduced and allow exploration of any performance bias affecting validity of the trial comparison.

METHODS

We first elicited and mapped usual services available locally at each trial sites (each site comprised collaborative partnerships between National Health Service (NHS) organisations and local authorities), and second enumerated services accessed by participants in both trial arms.

Eliciting and mapping services
A mapping tool was drafted using an Excel worksheet following discussion within the research team. This sought to identify services available for pregnant teenagers and young families across seven initial domains: midwifery, health visiting (specialist public health nurses), education, housing, social care and other services (eg, Children's Centres) and funding schemes specifically for young parents). The tool was piloted with local coordinators at three sites who described service characteristics (e.g., provider, eligibility criteria) and were debriefed by telephone interview to assess feasibility. An amended version, which incorporated completion instructions (Appendix 1), a worked example and study information, was circulated via email to site contacts across health and social care nominated by local FNP project leads. . Site contacts were encouraged to engage heads of services and other local professional staff (e.g. housing support workers) to provide detail about specific services or domains and to provide documentary details on services if available. Respondents were asked to provide details of "routinely provided services within their local authority which may be provided to young women, but may be especially relevant to pregnant teenagers".

Summative content analysis was used to identify missing or incomplete data in submitted returns, and followed up if necessary.[9] Data were analysed thematically by researchers who also involved service experts to review the developing coding framework, which was then applied to the data using NVivo 8.[10]

For peer review only - http://bmjopen.bmj.com/site/about/guidelines.xhtml

c:\programdata\activepdf\dc_ent\tmp\6c500b\27691771_file000002_639940258.docx

A second round of online data collection addressing the same domains aimed to consolidate and confirm information already provided and to reduce variation that may be solely attributable to reporting bias.

Enumerating service by trial participants

Access to supportive services within each core domain was measured in the trial's follow-up telephone interview schedule at late pregnancy, and 6, 12 and 18 months postpartum.[7] These included use of childcare, primary (eg, midwifery, GP, health visiting) and secondary (eg, A&E, Out-patient, In-patients) healthcare attendances, sexual health (contraceptive services), formal education, Connexions, support with housing, and a range of additional support services. At 24 months additional questions asked about financial support.

Some data informed the separately reported cost-effectiveness analysis.[11] In the current analysis we describe the pattern of core service usage (eg, health visiting, midwifery, housing) for those in both trial arms, and the level of support provided additionally via FNP (for FNP clients, the Healthy Child Programme was delivered by FNs rather than by health visitors). Data on the latter were provided via the FNP national unit's Information System. Use of services was analysed descriptively and is reported by service domain showing counts and proportions for those in the two trial arms separately. Multivariable logistic regression was used to explore whether certain maternal characteristics were associated with level of observed HV support. Univariable association were screened using a $p<0.10$ cut off and retained in the final multivariable model. Estimates are shown as odds ratios (ORs) and 95% confidence intervals (CIs).

RESULTS

Eliciting and mapping services

In round one, all sites responded, with at least six individual informants contributing data per site. A varying level of detail was provided about identified services. In general spreadsheets circulated to multiple stakeholders were more comprehensively completed.

Similar services within any one domain were subsequently grouped together even if labelled differently by informants. This resulted in 161 identified services, some with similar aims. An example was that of education provided to pregnant teenagers aged under 16 years old with eight different named services. In round two, the 161 services were listed, categorised into 12 service domains (the original domains plus 'other services' sub-divided on the basis of stage 1 responses into childcare, complex needs, Connexions, drug and alcohol, mental health, third sector, and sexual health).

The total number of services identified per site ranged from 52 to 113. These included between 26 and 53 universal services and between 22 and 86 locally available / specialist services. Services were provided by public, private and third sector organisations and collectively delivered direct care, support or guidance. Examples of Specialist and Locally available services are shown in table 1.

For peer review only - http://bmjopen.bmj.com/site/about/guidelines.xhtml

c:\programdata\activepdf\dc_ent\tmp\6c500b\27691771_file000002_639940258.docx

Table 1: Service mapping – examples of services[1] described by study sites[3]

Domain	Specialist services - specifically for pregnant teenagers or younger parents	Locally Available services - with a specialist nature and eligibility criteria, but not necessarily designed for teenage parents
**Education**	• Schools / colleges with provision for teenage mums<br>• Teenage pregnancy support services<br>• Accredited courses with free child care for under 25s	• Home learning programmes
**Housing**	• Teenage parents' scheme: training in independent living skills<br>• Supported housing: young vulnerable women or teenage parents	• Outreach support service aimed at young homeless people under 18<br>• Mother and Baby Hostel
**Health Visiting**		• Antenatal contact at home or in midwife-led antenatal clinics<br>• Minor ailments sessions run by health visitors
**Midwifery**	• Teenage pregnancy midwives<br>• Antenatal clinics run by midwives in schools	• Midwives based in Children's Centres
**Social Services**	• Teenage pregnancy support service	• Targeted youth support for vulnerable young people<br>• Specialist therapeutic unit for young victims of sexual abuse<br>• Family resource service; practical support to access universal services
**Connexions Services[2]**	• Teenage Pregnancy Advisors help young mums-to-be and young families	• Provide information and guidance to Looked After young people<br>• Provide support and guidance for young people leaving care<br>• Provide practical help and advice for young mums who want to go back to college
**Drugs, Alcohol and Smoking**		• Specialist drugs and alcohol services working with police<br>• Community-based young people's drugs and alcohol service<br>• Smoking in Pregnancy cessation service
**Sexual Health**	• Lifestyle services working with teenage parents to prevent second pregnancy	• Family planning services for under 25-yr-olds in community settings<br>• Sexual health services for teenagers<br>• Condom distribution scheme in community settings

For peer review only

c:\programdata\activepdf\dc_ent\tmp\6c500b\27691771_file000002_639940258.docx

**Mental Health services**		• Specialist Children's and Adolescent Mental Health Services for eating disorders<br>• Mother-and-baby units in hospitals and prisons<br>• Specialist psychiatric unit for postnatal mental illness
**Complex Needs services**<br>**Childcare provision**	• Support and advocacy for (pregnant) teenagers with complex needs	• Child development centre for pre-school children with complex needs<br>• Sure Start language therapy team<br>• Vulnerable baby service: targeted safeguarding prevention<br>• Private, voluntary, independent childcare providers<br>• Internet database on county-wide childcare provision
**Local / third sector projects**	• Charity funded teen parents projects<br>• Peer support sessions for teenage fathers-to-be	• Barnardo's Priory Family Centre<br>• Charity funded young parents projects<br>• Home Start: trained volunteers visit mums for approx. 15 months

1 Set information provided by local informants for each reported service included: Name of service, Narrative description, Limits on availability (eg, upper limit on number of women offered service, Location (eg, base), Level of service provision per client (eg, frequency, duration, quantity), Illustrative current caseload, Delivery setting, Client eligibility criteria, Service provider (eg, local authority), Assessment of local service variations compared to other locations
2 A government funded advisory and support service for young people aged 13-19 years old, now discontinued
3 Data collection timing: Round 1 - Data collection was requested over a six-week period from August 2009 to coincide with early stages of trial recruitment; *Round 2:* The survey link was sent to local FNP supervisors for completion in July 2011.

For peer review only

For peer review only - http://bmjopen.bmj.com/site/about/guidelines.xhtml

c:\programdata\activepdf\dc_ent\tmp\6c500b\27691771_file000002_639940258.docx

Not all universal services were reported from all sites although these would have been available (eg, universal education provision to age 16 years). In the domains of mental health, addiction and complex needs provision a small number of sites reported no additional locally available or specialist services. No sites reported specialist health visiting services for teenagers. Fourteen sites reported the employment of specialist teenage pregnancy midwives. Details from local informants describe the type and range of services available across the range of providers and sector domains. Services were numerous, complex and in some cases with fluid boundaries facilitating multi-disciplinary interaction to support users. Individual services although provided with similar intent could vary by site, while administrative boundaries between services were shown to be fluid.

Service usage during the trial

Initially 823 women were allocated to receive FNP and 822 women to Usual Care (UC) and following mandatory or elective withdrawal (including of consent), 808 and 810 women respectively completed baseline assessment.[8] Interviews were completed with 501 women (FNP) and 466 women (UC) at 18 months. At 24 months follow-up the number of interviews completed were 595 (FNP) and 559 (UC).

Community health visiting, midwifery and FNP

Core publicly funded services for mothers are maternity care and health visiting. The mean number of all home visits from health visitors was similar in both study arms (UC: 5.01 (SD 5.51); FNP: 4.70 (SD 7.81)). Contact with health visitors in clinic was quite different with more reported by mothers in the UC arm (mean 6.31, SD 7.07) than in the FNP arm (0.70, SD 2.92). The number of contacts within each reporting period up to 18 months reflects a similar pattern (table 2). The mean number of community midwifery contacts during pregnancy for the 422 UC women responding in late pregnancy was 10.69 (SD: 5.34) and for the 459 in the FNP arm was 10.68 (SD: 5.25). Women allocated to FNP received an average of 9.71, 18.63 and 13.22 valid FN visits per programme phase (Pregnancy, Infancy, Toddlerhood) with average visit duration of 79.14 minutes. There was a programme attrition rate by phase of 3.6%, 10.1% and 7.9% respectively (cumulative rate of 21.1%).

For peer review only

c:\programdata\activepdf\dc_ent\tmp\6c500b\27691771_file000002_639940258.docx

Table 2 Participant reported access to health services (health visitor and contraception) by follow-up (month)

	6		***12***		***18***		**Combined (up to 18 months)**	
	FNP n=511	*UC n=470*	*FNP n=514*	*UC n=483*	*FNP n=501*	*UC n=466*	*FNP n=501*	*UC n=466*
**Health visitor contacts** *Mean (SD)*								
Home	3.07 (6.08)	3.35 (3.58)	1.24 (3.67)	1.16 (2.63)	0.50 (2.50)	0.93 (2.58)	4.70 (7.81)	5.01 (5.51)
Clinic	0.51 (2.12)	3.72 (5.04)	0.20 (1.37)	1.66 (2.76)	0.06 (0.45)	1.01 (2.51)	0.70 (2.92)	6.31 (7.07)
**Contraceptive services** *%*								
GP surgery	42.3	38.3	41.2	44.1	38.5	46.1		
Family planning clinic	26.2	19.8	19.6	18.6	22.6	18.7		
Children's centre	1.4	0.6	1.0	0.8	1.0	0.4		
Sexual health clinic	6.1	4.5	4.7	4.3	7.2	4.5		

We explored variation in core service usage to determine whether level of observed support (</=3 HV home visits / 3+ HV home visits) was directed to participants distinguishable on the basis of baseline characteristics (Table 3). Women who had ever been homeless, had a higher subjectively defined social status, and poorer mental health were associated with 4 or more visits, while visit frequency also varied by trial site (but was not subsequently entered into the final model) (table 3). Homelessness (OR=1.80, 95% CI=1.02 to 3.17) and subjective social status (OR=1.13, 95% CI=1.01 to 1.27) were the only two individual characteristics that remained independently associated with visit numbers.

For peer review only - http://bmjopen.bmj.com/site/about/guidelines.xhtml

c:\programdata\activepdf\dc_ent\tmp\6c500b\27691771_file000002_639940258.docx

Table 3 Baseline predictors of number of home visits from health visitor by six months postpartum for women in usual care arm

	3 or fewer visits (N=155) N	Median (IQR[a]) or %	4 or more visits (N=312) N	Median (IQR[a]) or %	Overall N=467) Median (IQR[a]) or N (%)	Univariate assoc.[g]
**Age in years**		17.9 (17.1 – 18.7)		17.8 (16.9 – 18.9)	17.8 (16.9 – 18.8)	0.721
**Ethnic background**						0.070
White	130	83.9	276	88.5	406 (86.9)	
Mixed	5	3.2	18	5.9	23 (4.9)	
Asian	3	1.9	4	1.3	7 (1.5)	
Black	15	9.7	13	4.2	28 (6.0)	
Other	2	1.3	1	0.3	3 (0.6)	
**Relationship status**						0.433
Married	4	2.6	3	1.0	7 (1.5)	
Separated	13	8.4	34	10.9	47 (10.1)	
Closely inv. / boyfriend	120	77.4	244	78.2	364 (77.9)	
Just friends	18	11.6	31	9.9	49 (10.5)	
**Live with father of baby**						0.512
Yes	42	27.1	71	22.8	113 (24.2)	
No	108	69.7	212	67.9	320 (68.5)	
Not answered	5	3.2	29	9.3	34 (7.3)	
**Subjective social status:**						
**Family**	155	5.8 (5.0 – 7.0)	309	5.8 (5.0 – 7.0)	5.8 (5.0 – 7.0)	0.896
**Personal**	154	6.8 (5.0 – 8.0)	311	7.1 (6.0 – 8.0)	6.7 (6.0 – 8.0)	**0.007[g]**
**NEET[b]**:	138		266			0.210
Yes	45	32.6	105	39.5	150 (37.1)	
No	93	67.4	161	60.5	254 (62.9)	
**Receive any benefits**	154		311			0.776
Yes	48	31.0	101	32.4	149 (31.9)	
No	106	68.4	210	67.3	316 (67.7)	
Not answered	1	0.6	1	0.3	2 (0.4)	
**Ever been homeless**						**0.023[g]**
Yes	19	12.3	65	20.8	84 (18.0)	
No	136	87.9	247	79.2	383 (82.0)	
**Deprivation (IMDS)[c]**	154	40.4 (24.8 – 54.3)	308	38.0 (24.8 – 51.4)	38.8 (24.8 – 51.7)	0.175
**Health utility**						0.374
Perfect health	104	67.1	195	62.5	299 (64.0)	
Less than perfect health	51	32.9	115	36.9	166 (35.5)	
Not answered	0	0.0	2	0.6	2 (0.4)	
**Self-rated health**						0.227
Excellent	24	15.5	58	18.6	82 (17.6)	
Good	113	72.9	200	64.1	313 (67.0)	
Fair	17	11.0	48	15.4	65 (13.9)	
Poor	1	0.6	6	1.9	7 (1.5)	
**Limiting chronic illness**:						0.144
Yes	24	15.5	66	21.2	90 (19.3)	
No	131	84.5	246	78.8	377 (80.7)	

For peer review only - http://bmjopen.bmj.com/site/about/guidelines.xhtml

c:\programdata\activepdf\dc_ent\tmp\6c500b\27691771_file000002_639940258.docx

**Self-efficacy**[d]	151	29.7 (27.0 – 32.5)	308	29.9 (28.0 – 32.0)	29.8 (27.0 – 32.0)	0.604
**Adaptive functioning:** [e] **Difficulty in at least one basic skill**						0.674
Yes	36	23.2	78	25.0	114 (24.4)	
No	119	76.8	234	75.0	353 (75.6)	
**3 or fewer key life skills**						0.822
Yes	39	25.2	81	26.0	120 (25.7)	
No	116	74.8	229	73.4	345 (73.9)	
Missing	0	0.0	2	0.6	2 (0.4)	
**At least one burden**						0.080
Yes	55	35.5	87	27.9	142 (30.4)	
No	98	63.2	224	71.8	322 (69.0)	
Missing	2	1.3	1	0.3	3 (0.6)	
**Alcohol / drug use** [f]	147	1.2 (0.0 – 2.0)	296	1.3 (0.0 – 2.0)	1.3 (0.0 – 2.0)	0.212
**Antisocial behaviour**	154	2.0 (1.0 – 3.0)	310	2.3 (1.0 – 4.0)	2.2 (1.0 – 3.0)	0.088
**Social support**	155	85.7 (77.0 – 98.7)	310	85.8 (79.0 – 98.7)	85.8 (77.6 – 98.7)	0.491
**Relationship quality**	130	28.5 (26.0 – 32.0)	255	28.2 (26.0 – 32.0)	28.3 (26.0 – 32.0)	0.433
**Family resources**	150	13.5 (11.0 – 16.0)	296	13.5 (11.0 – 16.0)	13.5 (11.0 – 16.0)	0.884
**Psychological distress / Mental health**	155	20.3 (15.0 – 25.0)	311	21.8 (17.0 – 26.0)	21.3 (16.0 – 26.0)	0.025
**Trial site**						0.003[h]
1	1	0.6	10	3.2	11 (2.4)	
2	5	3.2	8	2.6	13 (2.8)	
3	14	9.0	15	4.8	29 (6.2)	
4	2	1.3	7	2.2	9 (1.9)	
5	8	5.2	10	3.2	18 (3.9)	
6	6	3.9	7	2.2	13 (2.8)	
7	7	4.5	7	2.2	14 (3.0)	
8	12	7.7	19	6.1	31 (6.6)	
9	13	8.4	26	8.3	39 (8.4)	
10	5	3.2	17	5.4	22 (4.7)	
11	7	4.5	30	9.6	37 (7.9)	
12	17	11.0	16	5.1	33 (7.1)	
13	7	4.5	35	11.2	42 (9.0)	
14	5	3.2	3	1.0	8 (1.7)	
15	11	7.1	26	8.3	37 (7.9)	
16	19	12.3	19	6.1	38 (8.1)	
17	8	5.2	30	9.6	38 (8.1)	
18	8	5.2	27	8.7	35 (7.5)	

a Interquartile range; b Definition of NEET: Not in education employment or training (applicable only to those whose academic age is >16 at baseline interview); c Higher IMD score indicated more deprivation; d Higher score indicates higher level of self-efficacy; e Higher score indicates better management of day-to-day lives and routines (for each of the three sub-scales); f CRAFFT screening test[12] for substance related risks and problems in adolescents; g italics indicate variable included in logistic regression, bold indicates variable remained significantly associated with number of visits in logistic model; h not modelled in regression analysis due to high number of levels

Other services

For peer review only - http://bmjopen.bmj.com/site/about/guidelines.xhtml
For peer review only

Participants accessed a wide range of services encompassing housing support, financial support, education, healthcare, childcare and social care (tables 4 to 5). A small proportion of respondents reported accessing support for housing outside of their friends and family, mostly from the local authority (table 4). The small difference in reported rates between study arms would appear to have been in part attributable to additional assistance from the FNP family nurse. Most participants reported being in receipt of additional publicly funded financial support. For most participants this included income support, housing benefit and council tax reductions with similar rates between study arms reported. Smaller proportions of participants reported other forms of financial assistance related to employment, education or personal health (eg, Jobseekers allowance). The largest difference in reported rates between study arms was for those who received regular financial support from parents: 8.9% (FNP), 15.4% (UC).

Table 4 Proportion of participants reporting housing and financial support by follow-up point (months)

	6		12		18		24	
	FNP n=511	*UC n=470*	*FNP n=514*	*UC n=483*	*FNP n=501*	*UC n=466*	*FNP n=595*	*UC n=559*
i) Source of housing support								
Anyone outside of friends or family	18.0	14.9	12.1	9.9	9.2	8.4	12.1	9.7
Local authority housing department	7.0	6.6	5.1	5.6	4.6	4.7	6.2	5.9
Family Nurse	4.1	-	3.1	-	2.2	-	5.4	-
ii) Source of financial support								
State benefits or payments	-		-		-		86.9	88.4
Income support	-		-		-		62.0	63.3
Jobseekers allowance	-		-		-		8.6	8.9
Housing benefit	-		-		-		64.2	68.5
Council tax reduction	-		-		-		62.9	63.3
Disability living allowance	-		-		-		2.5	5.4
Incapacity benefit	-		-		-		0.7	1.6
Child Support Agency[1]	-		-		-		12.8	11.6
Regular support from parents	-		-		-		8.9	15.4
Education grants	-		-		-		5.5	5.9

1 Directly or via partner

Most women seeking contraception obtained it from their general practice, and to a lesser extent from a family planning clinic. There were some small differences between study arms by time point (eg at 18 months 46.1% of women in the UC arm accessed contraception from their GP, while 38.5% in the FNP did) but overall use of this service was similar. The proportion of women accessing any education gradually increased across the duration of the trial. By 24 months about a fifth of women were in school, college or training (FNP: 22.5%, UC: 18.1%). This was mostly in mainstream education, although there were a small number of women in both trial arms accessing support in more specialised units (eg, learning support unit). A similar pattern of increasing support for childcare was observed over time with approximately a quarter of women reporting some form of childcare support used at 24 months. Support was received from a variety of sources and there appeared to be a similar pattern of usage between study arms.

For peer review only - http://bmjopen.bmj.com/site/about/guidelines.xhtml

c:\programdata\activepdf\dc_ent\tmp\6c500b\27691771_file000002_639940258.docx

Table 5 Proportion of participants reporting access to education, childcare and other support services by follow-up point (months)

	6		12		18		24	
	FNP n=511	*UC n=470*	*FNP n=514*	*UC n=483*	*FNP n=501*	*UC n=466*	*FNP n=595*	*UC n=559*
i) Education attended								
Any school, college or training	14.5	16.4	20.4	19.0	22.4	20.6	22.5	18.1
Mainstream school or college	11.3	13.7	15.0	15.6	19.5	18.7	16.6	12.7
Learning support unit	0.6	0.2	0.6	0.6	0.2	0	0.7	0.7
Pupil referral unit	0	0.2	0	0	0	0	0	0.2
Teenage mums support unit	0.8	1.7	0.6	0.6	0.4	0.6	0.7	1.5
ii) Childcare accessed								
Any childcare	7.0	7.0	16.1	13.3	25.5	21.5	26.9	24.3
Crèche at school or college	4.1	4.5	8.8	6.6	4.8	3.6	12.1	12.3
Day nursery at children's centre	0.8	0.6	0	0	3.6	2.4	5.5	4.3
Child-minder	1.8	1.1	2.1	1.2	3.2	2.4	3.2	3.0
Other forms of childcare	0.8	0.6	2.1	2.9	8.0	6.9	6.7	6.1
iii) Other support services								
Connexions	31.1	26.8	23.5	23.2	16.8	17.0	*	*
School nurse	1.4	1.5	0.8	0.4	0	0.9	0.5	0.9
Young People's Centre	4.9	7.0	2.7	3.9	1.8	1.9	1.8	1.6
Family Information Centre	2.0	2.3	1.2	1.5	2.2	3.0	1.3	1.4
Children's Centre	36.6	36.6	25.8	35.6	28.3	30.0	34.6	26.7
Child development centre	0.6	0.6	0.4	1.7	0.8	1.5	1.0	2.5
Crèche/ day nursery	10.8	10.8	15.4	14.7	8.4	6.0	17.6	16.6
Toddler group	7.8	7.9	12.5	11.0	16.2	15.2	19.2	21.5
Leaving care service	1.4	0.4	1.8	1.0	1.4	0.6	2.0	0.9
Fostering service	0.6	0.2	0.4	0.4	0	0.6	0.3	0.4
Youth offending team	0.8	0.9	0.2	0.2	0.4	0	0.3	0
Social worker	10.6	10.0	7.4	7.5	8.2	6.2	13.1	9.7
Alcohol / drug support	0.6	0	0.2	0.2	0	0.4	0.3	0.5

1 Some respondents indicated they were in school, college or training but provided no further information
** Not collected as service reconfigured*

Various other services were accessed, the most frequent being Connexions and Children's Centres. The former was used with decreasing frequency over time (consistent with the aging profile of the sample), while the latter showed a more variable pattern of access across each time point and on occasions quite different rates of access between trial arms. At six months one in ten mothers in both trial arms reported contact with a social worker, a rate that varied over time to 24 months at which point there was only a small difference between groups (FNP: 13.1%, UC: 9.7%).

DISCUSSION

To understand the service context within which FNP was trialled we mapped the range of services available. The multiplicity of services often within the same area and their varying labels often concealed similarities and differences between services. We established the usage of key services by trial participants across service domains. We particularly focused on those most directly relevant to the intervention (eg, health visiting) although included many other services. With mostly only small differences in usage between trial arms perhaps what is most important is the wide range of services being accessed. Although the previous US

For peer review only - http://bmjopen.bmj.com/site/about/guidelines.xhtml

trials have not further reported on broader services, the contexts were likely to be very different from the English trial setting.

FNP aims to impact upon a range of maternal and child outcomes. Therefore our selection of relevant services was necessarily broad and informed by the intervention's theory of change, which includes promoting access to services. However, previously reported attempts to map services have been challenging even when restricted to a single organisation.[13] To cope with such complexity researchers have sought to distinguish between specialist and generic services, including through a multi-staged approach as used here.[14] It has been consistently reported that information provision is time consuming in such exercises, as we also found.[15] Individual informants may be unfamiliar with all relevant services even within their professional area, hence the coordinated approach to data gathering from multiple informants we used. Feedback from FNP staff in our process evaluation focus groups highlighted a similar challenge when acquiring knowledge about local services, essential for then linking up clients to relevant support.[11] Some core services such as mainstream education were not always reported and illustrates the need to clearly define the scope of the information request to informants, especially the boundaries within which they are being asked to respond.

In effectiveness trials existing services could respond by augmenting support to those in the control arm. Such performance bias limits generalizability especially if that support was very different from usual care and approaching the level of support provided by the new intervention. Our findings do not indicate this in general and specifically for community midwifery and health visiting, the two most closely aligned universal services. Community midwives visits were equivalent between trials arms and the difference in contacts with health visitors was attributable to clinic rather than home visits and therefore unlikely to be substantial. There was some indication that women in the usual care arm with some additional objective need identified at baseline, such as experience of homelessness, received more home visits. However, providing enhanced care to clients most in need would be usual practice. Evidence that this occurred in a trial context is not in itself a threat to external validity. The large caseloads managed by health visitors emphasises the lack of opportunity to provide significant additional support to mothers allocated to usual care.[11]

Our trial found fewer short-term benefits than previous US trials despite FNP being well implemented.[1-3, 8] The population studied differed from that in the US, for example by being fundamentally identified by maternal age and this may have contributed to some differences in impact detected. However, the service context would have been very different. Some additional standardised support in the form of developmental screening and referral, and free travel to appointments was provided to women in the control arm of each US trial. However, the broad and layered range of services identified in our study would not have been available. The extreme community disadvantage present in the first US trial from which much longer-term evidence has accrued (at the inception of the first US trial Elmira was ranked bottom of all 380 metropolitan statistical areas in terms of economic conditions) additionally compounds this. That is not to say that women in our trial were free of disadvantage or had services that fully met their needs. However, substantial differences across trial settings are likely to have varied the potential for beneficial impact.

Service provision may change over time and any single mapping exercise will miss this real-world dynamic. Quantification of service use should be open to the capture of newer services. Additionally, with superficial service names not always reflecting well actual support provided it is important to look beyond service labels. Finally, high-level service descriptions do not always represent the often complex multi-professional interactions which necessarily facilitate service delivery. This emphasises the need for adequate qualitative description and interpretation of services.

Loss to follow-up at assessment points may introduce bias into the descriptive analysis. We have previously reported on group differences in attrition apparent at 24 months follow-up however such, differences were small.[11] A second consideration is the level of detail available for health visitor and midwifery contacts (eg, visit duration). It is reasonable to assume that given capacity and opportunity, women in the UC arm visited by health professionals would have received greater attention than other clients perceived as less in need. This is consistent

For peer review only - http://bmjopen.bmj.com/site/about/guidelines.xhtml

c:\programdata\activepdf\dc_ent\tmp\6c500b\27691771_file000002_639940258.docx

with their professional role and reflective of contemporary best usual practice.[16] It is also possible that women in the FNP arm received relatively less attention than non-FNP clients if they were seen to be receiving enhanced support. Nevertheless, the total number of home and clinic visits received in both trial arms was small compared to that provided by FNP nurses. Future process evaluations should model the impact upon existing services of such service innovation to both avoid unintended consequences (eg, service displacement) and maximise synergy across services.

The effectiveness of a public health intervention can only be adequately evaluated with a sound understanding of the service context within which it operates and which may also form the trial comparator.[17] Describing and quantifying the nature of usually available services can be challenging especially when services arise from a number of sectors, may evolve over the period of study and vary across study sites. In mapping the pattern of support potentially available to participants in our trial we have gained a critical understanding of the context within which and against which FNP should be considered. While challenging, we remain convinced of the need to develop this area of research when evaluating public health interventions. Indeed, in their feedback survey respondents reported the usefulness of the exercise in gaining greater insights about local services, some sharing the generated service summaries with their teams. Finally, we quantified maternal reported service usage to provide key insights into how our main trial results should be interpreted.

For peer review only - http://bmjopen.bmj.com/site/about/guidelines.xhtml

For peer review only

c:\programdata\activepdf\dc_ent\tmp\6c500b\27691771_file000002_639940258.docx

ACKNOWLEDGEMENTS

The authors gratefully acknowledge the contribution of Dr Lesley Wye, University of Bristol and Dr Marie-Jet Bekkers who contributed to some of the planning and implementation of the reported study, and to all other members of the broader Building Blocks research team who supported overall trial delivery. We are grateful to each local site informant who contributed to data collection in either of the two rounds of service mapping and especially to the three local coordinators who initially piloted our mapping tool. We are also grateful to all women who participated in the trial and for their time in completing outcome and resource assessments.

COMPETING INTERESTS

Prior to working on the Building Blocks trial, Sue Channon facilitated two workshops on Motivational Interviewing for supervisors within the FNP. The authors declare no competing interests.

CONTRIBUTORS

MR, KH and JS conceived the study and all authors contributed to the development of its protocol. MR wrote the first draft with further contributions from all authors. RP, JS and GM were involved in data collection and management. RP was responsible for developing the survey of local stakeholders and GM was responsible for managing data collected from trial participants used in the analysis. RP, RCJ, GM and MR were involved in analysis and developing summary tables for publication. SC and JS were responsible for the management of this package of work within the trial overall. MR was responsible for obtaining study funding. All authors contributed to data interpretation, reviewed successive drafts and approved the final version of the manuscript.

ETHICS

The trial was approved by the Wales NHS Research Ethics Committee (09/MRE09/08) and received governance approval from all participating NHS sites. All women provided written informed consent.

PARTICIPANT CONSENT

All participants provided written informed consent to take part in the study.

FUNDING

The South East Wales Trials Unit (SEWTU), Centre for Trials Research, Cardiff University is funded by the Welsh Government through Health and Care Research Wales and the authors gratefully acknowledge SEWTU's contribution to study implementation. This is an independent report commissioned, sponsored and funded by the Policy Research Programme in the Department of Health (reference no. 006/0060). The views expressed are not necessarily those of the Department of Health.

DATA SHARING

The datasets generated and analysed during the current study are not publicly available as contributors / participants may be identifiable and are also subject to sponsor approval, but may be available from the corresponding author on reasonable request.

For peer review only

For peer review only - http://bmjopen.bmj.com/site/about/guidelines.xhtml

c:\programdata\activepdf\dc_ent\tmp\6c500b\27691771_file000002_639940258.docx

References

1. Olds DL, Henderson CR, Jr., Chamberlin R, Tatelbaum R. Preventing child abuse and neglect: a randomized trial of nurse home visitation. Pediatrics 1986; 78 (1): 65-78.
2. Kitzman H, Olds DL, Henderson CR, Jr., et al. Effect of Prenatal and Infancy Home Visitation by Nurses On Pregnacy Outcomes, Childhood Injuries, and Repeated Childbearing: A Randomised Controlled Trial. JAMA 1997; 278 (8): 644-52.
3. Olds DL, Robinson J, O'Brien R, et al. Home Visiting by Paraprofessionals and by Nurses: A Randomized, Controlled Trial. Pediatrics 2002; 110 (3): 486-96.
4. Barnes J, Ball M, Meadows P et al. Nurse-Family Partnership Programme: First Year Pilot Sites Implementation in England - Pregnancy and the Post-partum Period. London: Birkbeck, University of London; 2008.
5. Barnes J, Ball M, Meadows P et al. Nurse-Family Partnership Programme: Second Year Pilot Sites Implementation in England - The Infancy Period. London: Birkbeck, University of London; 2009.
6. Barnes J, Ball M, Meadows P, et al. The Family-Nurse Partnership Programme in England: Wave 1 implementation in toddlerhood & a comparison between Waves 1 and 2a of implementation in pregnancy and infancy. London: Institute for the Study of Children, Families and Social Issues: Birkbeck, University of London, 2011.
7. Owen-Jones E, Bekkers M-J, Butler CC, et al. The effectiveness and cost-effectiveness of the Family Nurse Partnership home visiting programme for first time teenage mothers in England: a protocol for the Building Blocks randomised controlled trial. BMC Pediatrics 2013; 13 (1): 114.
8. Robling M, Bekkers M-J, Bell K, et al. Effectiveness of a nurse-led intensive home-visitation programme for first-time teenage mothers (Building Blocks): a pragmatic randomised controlled trial. Lancet 2015; published online Oct 14. http://dx.doi.org/10.1016/S0140-6736(15)00392-X.
9. Hsieh HF, Shannon SE. Three approaches to qualitative content analysis. Qualitative Health Research 2005 15: 1277-88
10. NVivo qualitative data analysis Software; QSR International Pty Ltd. Version 8, 2008
11. Robling MR Bekkers M-J, Bell K et al. Evaluating the Family Nurse Partnership Programme in England: A Randomised Controlled Trial. Final report to The Department of Health. 2015. (http://bit.ly/buildingblockstrial).
12. Knight JR, Sherritt L, Shrier LA et al Validity of the CRAFFT substance abuse screening test among adolescent clinic patients. Arch Pediatr Adolesc Med 2002, 156(6):607–614.[CRAFFT].
13. Hotopf M, Rayner L, Valsraj K. Improving ‘Whole Person Care’ A report on mental health related services offered by King’s Health Partners’ acute trusts: King's Health Partners, 2012.
14. Challis D, Darton R, Donnelly M et al. Mapping and evaluation of care management arrangements for older people and those with menatal health problems in an integrated health and social services system: a comparative study: The Personal Social Services Research Unit, 1999.
15. Health Do. Children's services mapping. A complete picture of children's service provision: Department of Health. Department for Children Schools and Families, 2010.
16. NICE. Pregnancy and complex social factors: A model for service provision for pregnant women with complex social factors. London, 2010.
17. Smelt AFH, van der Weele GM, Blom JW et al. How usual is usual care in pragmatic intervention studies in primary care? An overview of recent trials. British Journal of General Practice 2010 e305.

For peer review only

BMJ Open

What is usual care for teenagers expecting their first child in England?: a process evaluation using key informant mapping and participant survey as part of the Building Blocks randomised controlled trial of specialist home visiting.

Journal:	*BMJ Open*
Manuscript ID	bmjopen-2017-020152.R1
Article Type:	Research
Date Submitted by the Author:	28-Dec-2017
Complete List of Authors:	Robling, Michael; Cardiff University, Centre for Trials Research<br>Cannings John, Rebeca; Cardiff University, Centre for Trials Research<br>Channon, Sue; Cardiff University, Centre for Trials Research<br>Hood, Kerenza; Cardiff University, South East Wales Trials Unit, Centre for Trials Research<br>Moody, Gwenllian; Cardiff University, Centre for Trials Research<br>Poole, Ria; Cardiff University, Medicine<br>Sanders, Julia; School of Healthcare Sciences, Room 12:15, Eastgate House, 35-43 Newport Road
**Primary Subject Heading**:	Public health
Secondary Subject Heading:	Public health, Qualitative research, Research methods
Keywords:	Organisation of health services < HEALTH SERVICES ADMINISTRATION & MANAGEMENT, PAEDIATRICS, Child protection < PAEDIATRICS, PUBLIC HEALTH

SCHOLARONE™
Manuscripts

For peer review only - http://bmjopen.bmj.com/site/about/guidelines.xhtml

c:\programdata\activepdf\dc_ent\tmp\456441\28623087_file000006_653285914.docx

Title: What is usual care for teenagers expecting their first child in England?: a process evaluation using key informant mapping and participant survey as part of the Building Blocks randomised controlled trial of specialist home visiting.

Corresponding author:

Prof Mike Robling
Centre for Trials Research, Cardiff University
Neuadd Meirionnydd, Heath Park, Cardiff. CF14 4YS
roblingmr@cardiff.ac.uk
+44 (0)29 2068 7177

Authors:

- Mike Robling, Centre for Trials Research, Cardiff University, Cardiff, Wales, UK
- Rebecca Cannings-John, Centre for Trials Research, Cardiff University, Cardiff, Wales, UK
- Sue Channon, Centre for Trials Research, Cardiff University, Cardiff, Wales, UK
- Kerry Hood, Centre for Trials Research, Cardiff University, Cardiff, Wales, UK
- Gwen Moody, Centre for Trials Research, Cardiff University, Cardiff, Wales, UK
- Ria Poole, School of Medicine, Cardiff University, Cardiff, Wales, UK
- Julia Sanders, School of Healthcare Sciences, Cardiff University, Cardiff, Wales, UK

Key words: Public health, child health, nursing, randomised trial

Current abstract word count: 299
Current manuscript word count: 4080
Reference count: 19

For peer review only

c:\programdata\activepdf\dc_ent\tmp\456441\28623087_file000006_653285914.docx

Title: What is usual care for teenagers expecting their first child in England?: a process evaluation using key informant mapping and participant survey as part of the Building Blocks randomised controlled trial of specialist home visiting.

Abstract

Objectives
We compared the US-derived Family Nurse Partnership (FNP) home visiting programme when added to usually provided health and social care for first-time teenage mothers, to usual care alone. We aimed to: establish the nature of usual care, measure service usage and assess performance bias in core usual care services.

Design
Within trial process evaluation. Local professionals completed a survey mapping local health and social care services in seven domains. This focused on services available to young women, especially those relevant to pregnant teenagers. Descriptive data were assessed thematically to establish the range of services. Quantitative data collection with FNP supervisors enumerated service provision by site. Services identified were included in main participant trial follow-up interviews at four time-points to quantify usage. Usage was described descriptively by domain. We explored predictors of health visitor visits.

Setting
Eighteen partnerships of local authority and healthcare organisations in England.

Outcomes
Descriptive framework of services. Rates of service usage reported by trial participants.

Results
161 separate services were identified, with multiple service models in each domain, broadly categorised as universal or specialist (eg for teenage mothers). FNP supervisors identified 30-63 universal services per site and 22-67 specialist services. Use of core maternity care services were similar across trial arms and with only small differences in use of health visiting services. Participants accessed a wide range of services. Women who had ever been homeless, who had a higher subjectively defined social status, and poorer mental health received more visits from a health visitor.

Conclusions
The large number of services available to teenage mothers in England may limit the incremental benefit achievable through enhanced home visiting. There was little evidence of compensatory practice, such as additional care for women in the usual care arm. Measuring usual care when trialling complex interventions is challenging and essential.

For peer review only

c:\programdata\activepdf\dc_ent\tmp\456441\28623087_file000006_653285914.docx

Strengths and limitations of this study

- The identification of sometimes multiple local stakeholders and drawing upon their existing knowledge using a semi-structured self-completion tool about a range of relevant services enabled us to develop a rich picture of what may be usually available care for teenagers expecting their first child.
- Undertaking the initial mapping exercise enabled us to develop a more informed service use inventory with greater content validity than may otherwise have been possible.
- The combination of professionally-led key informant mapping and detailed service use recording as part of trial follow-up data collection therefore provides a more nuanced understanding of usual care. This greater understanding of the trial's control condition enhances interpretation of trial results.
- However, changes over time, and within and between site differences in how services are configured, perceived and understood means that a summary statement about all locally relevant services will need to be intermittently revisited.
- Although we have an understanding about how services were similarly or differently accessed by intervention and control participants in the trial, the intensity and duration of individual sessions for non-FNP services is not known. However, comprehensively attempting to collect such detailed data from trial participants would probably not be feasible in practice
- Some bias due to participant attrition in follow-up interviews is likely despite relatively high trial retention rates, although other analyses show only minimal differences between those recruited and followed-up.

For peer review only

c:\programdata\activepdf\dc_ent\tmp\456441\28623087_file000006_653285914.docx

INTRODUCTION

Individual, social, and economic circumstances faced by teenage mothers can challenge a successful start for their children. Responding in 2006, the Government in England adopted a preventative US-derived programme of nurse-led intensive home-visiting, the Family Nurse Partnership (FNP). Specially trained family nurses support first-time mothers through up to 64 home visits starting in early pregnancy and until the child reaches their second birthday. In three US trials, the programme has been evaluated with differing socio-demographic populations, justifying initial testing in a UK context.[1-3]

Following an implementation evaluation, 18 English Primary Care Trust (PCT) sites participated in the Building Blocks trial (ISRCTN23019866) of the programme's effectiveness recruiting 1645 teenagers expecting their first child.[4-8] The sites were dispersed across the UK, and covered two rural and 16 city areas. Women were recruited before 25 weeks gestation, lived within geographical areas served by the FNP team and spoke at least conversational English. Assessing over 60 short-term outcomes (to 24 months post-partum) in domain areas of pregnancy and birth, child development, and maternal life-course, four primary outcomes of programme and policy interest were prioritised.

We compared FNP when added to usually provided health and social care to usual care alone. In the absence of comprehensive public healthcare in the US, across all three previous evaluations the counterfactual was reported as obstetric office-based antenatal care, paediatric developmental screening, referral at specified time points and free transport to office-based consultations. Elevating the control condition to just more than simply no care, the augmented control condition was not further described. Given the provision of free universal health services in the UK, the ethical trial comparator was an active control condition. However, it was expected that what would be available to young families may be complex and vary by site and over time.

We aimed to map and quantify usually provided care and so clarify the trial's control condition, the service context into which FNP was introduced and allow exploration of any performance bias affecting validity of the trial comparison.

METHODS

We first elicited and mapped usual services available locally at each of the 18 trial sites. each site comprised collaborative partnerships between National Health Service (NHS) organisations and local authorities. All sites had applied to the Department of Heath to be a provider of FNP including by demonstrating local clinical need and commitment to sustain local programme delivery. Sites included urban and rural settings across England and encompassed each of the ten strategic health authorities in England. Second, we enumerated services accessed by participants in both trial arms.

Eliciting and mapping services

A mapping tool was drafted using an Excel worksheet following discussion within the research team. This sought to identify services available for pregnant teenagers and young families across seven initial domains: midwifery, health visiting (specialist public health nurses), education, housing, social care and other services (e.g., Children's Centres) and funding schemes specifically for young parents). This would therefore include services that were also universally available, such as maternity care. The tool required the site contacts to provide the title of service and a brief description. It was piloted with local coordinators at three sites who described service characteristics (e.g., provider, eligibility criteria) and were debriefed by telephone interview to assess feasibility. An amended version, which incorporated completion instructions (Appendix 1), a worked example and study information, was circulated via email in the first instance to each site principal investigator (e.g., the local FNP project lead and in all cases not a member of the research team) who then cascaded to local contacts across health and social care (usually managers or heads of services). By engaging with heads of services and other local professional staff (e.g. housing support workers) further detail about specific services or domains were provided, including

For peer review only - http://bmjopen.bmj.com/site/about/guidelines.xhtml

c:\programdata\activepdf\dc_ent\tmp\456441\28623087_file000006_653285914.docx

documentation on local services where available. Respondents were asked to provide details of "routinely provided services within their local authority which may be provided to young women, but may be especially relevant to pregnant teenagers". In parallel with obtaining information describing available services, national policies and guidelines were sourced informing on the minimum expected standard of universally available services such as maternity care and state welfare (e.g. childcare vouchers). Mapping data were collected over six months.

Within sites and across respondents we reviewed submitted returns to identify missing or incomplete data (i.e., to identify the presence or absence of expected services / service descriptions) and followed up if necessary with local site contacts. This process was informed by documentary data provided by sites or available online. Data provided by sites were entered into NVivo 8[9] and analysed thematically by researchers who also involved service experts to review the developing coding framework before coming to a consensus on the final range of services available. A second round of online data collection addressing the same domains aimed to consolidate and confirm information already provided and to reduce variation that may be solely attributable to reporting bias. This comprised a structured form listing services by domain and tick boxes for respondents to indicate presence or absence. Free text ('Other') services allowed for unlisted services to be reported. Local FNP supervisors completed this form.

Enumerating service by trial participants

Trial participants were teenagers (aged 19 years or under at last menstrual period) expecting their first child, living in the catchment area for local FNP provision recruited before 25 weeks gestation, able to provide informed consent and competent to converse in English.[7] Access to supportive services within each core domain was measured as part of the trial's follow-up outcome evaluation telephone interview schedule at late pregnancy, and 6, 12 and 18 months postpartum.[7] These included use of childcare, primary (eg, midwifery, GP, health visiting) and secondary (eg, A&E, Out-patient, In-patients) healthcare attendances, sexual health (contraceptive services), formal education, Connexions (a government funded support and advisory service for young people aged 13-19 years old), support with housing, and a range of additional support services. At 24 months additional questions asked about financial support.

Some data informed the separately reported cost-effectiveness analysis.[10] In the current analysis we describe the pattern of core service usage (eg, health visiting, midwifery, housing) for those in both trial arms, and the level of support provided additionally via FNP (for FNP clients, the Healthy Child Programme was delivered by FNs rather than by health visitors). Data on the latter were provided via the FNP national unit's Information System. Use of services was analysed descriptively and is reported by service domain showing counts and proportions for those in the two trial arms separately. Multivariable logistic regression was used to explore whether certain maternal characteristics collected as part of the trial's baseline assessment were associated with level of observed HV support. We created a binary variable of number of HV visits which distinguished between a standard / expected level of care (less than 4 visits) and enhanced care (4 or more visits). Univariable association were screened using a p<0.10 cut off and retained in the final multivariable model. Estimates are shown as odds ratios (ORs) and 95% confidence intervals (CIs).

RESULTS

Eliciting and mapping services

round one was conducted in a six week period from August 2009. all sites responded, with at least six individual informants contributing data per site. A varying level of detail was provided about identified services. In general spreadsheets circulated to multiple stakeholders were more comprehensively completed.

Similar services within any one domain were subsequently grouped together even if labelled differently by informants. This resulted in 161 identified services, some with similar aims. An example was that of education provided to pregnant teenagers aged under 16 years old with

For peer review only - http://bmjopen.bmj.com/site/about/guidelines.xhtml

c:\programdata\activepdf\dc_ent\tmp\456441\28623087_file000006_653285914.docx

eight different named services. In round two, conducted in July 2011, the 161 services were listed, categorised into 12 service domains (the original domains plus ‘other services’ sub-divided on the basis of stage 1 responses into childcare, complex needs, Connexions, drug and alcohol, mental health, third sector, and sexual health).

The total number of services identified per site ranged from 52 to 113. These included between 26 and 53 universal services and between 22 and 86 locally available / specialist services. Services were provided by public, private and third sector organisations and collectively delivered direct care, support or guidance. Examples of Specialist and Locally available services are shown in table 1.

For peer review only

For peer review only - http://bmjopen.bmj.com/site/about/guidelines.xhtml

c:\programdata\activepdf\dc_ent\tmp\456441\28623087_file000006_653285914.docx

Table 1: Service mapping – examples of services[1] described by study sites[3]

Domain	Specialist services - specifically for pregnant teenagers or younger parents	Locally Available services - with a specialist nature and eligibility criteria, but not necessarily designed for teenage parents
**Education**	• Schools / colleges with provision for teenage mums<br>• Teenage pregnancy support services<br>• Accredited courses with free child care for under 25s	• Home learning programmes
**Housing**	• Teenage parents' scheme: training in independent living skills<br>• Supported housing: young vulnerable women or teenage parents	• Outreach support service aimed at young homeless people under 18<br>• Mother and Baby Hostel
**Health Visiting**		• Antenatal contact at home or in midwife-led antenatal clinics<br>• Minor ailments sessions run by health visitors
**Midwifery**	• Teenage pregnancy midwives<br>• Antenatal clinics run by midwives in schools	• Midwives based in Children's Centres
**Social Services**	• Teenage pregnancy support service	• Targeted youth support for vulnerable young people<br>• Specialist therapeutic unit for young victims of sexual abuse<br>• Family resource service; practical support to access universal services
**Connexions Services[2]**	• Teenage Pregnancy Advisors help young mums-to-be and young families	• Provide information and guidance to Looked After young people<br>• Provide support and guidance for young people leaving care<br>• Provide practical help and advice for young mums who want to go back to college
**Drugs, Alcohol and Smoking**		• Specialist drugs and alcohol services working with police<br>• Community-based young people's drugs and alcohol service<br>• Smoking in Pregnancy cessation service
**Sexual Health**	• Lifestyle services working with teenage parents to prevent second pregnancy	• Family planning services for under 25-yr-olds in community settings<br>• Sexual health services for teenagers<br>• Condom distribution scheme in community settings

For peer review only - http://bmjopen.bmj.com/site/about/guidelines.xhtml

c:\programdata\activepdf\dc_ent\tmp\456441\28623087_file000006_653285914.docx

**Mental Health services**		• Specialist Children's and Adolescent Mental Health Services for eating disorders<br>• Mother-and-baby units in hospitals and prisons<br>• Specialist psychiatric unit for postnatal mental illness
**Complex Needs services**	• Support and advocacy for (pregnant) teenagers with complex needs	• Child development centre for pre-school children with complex needs<br>• Sure Start language therapy team<br>• Vulnerable baby service: targeted safeguarding prevention
**Childcare provision**		• Private, voluntary, independent childcare providers<br>• Internet database on county-wide childcare provision
**Local / third sector projects**	• Charity funded teen parents projects<br>• Peer support sessions for teenage fathers-to-be	• Barnardo's Priory Family Centre<br>• Charity funded young parents projects<br>• Home Start: trained volunteers visit mums for approx. 15 months

1 Set information provided by local informants for each reported service included: Name of service, Narrative description, Limits on availability (eg, upper limit on number of women offered service, Location (eg, base), Level of service provision per client (eg, frequency, duration, quantity), Illustrative current caseload, Delivery setting, Client eligibility criteria, Service provider (eg, local authority), Assessment of local service variations compared to other locations
2 A government funded advisory and support service for young people aged 13-19 years old, now discontinued
3 Data collection timing: Round 1 - Data collection was requested over a six-week period from August 2009 to coincide with early stages of trial recruitment; R*ound 2:* The survey link was sent to local FNP supervisors for completion in July 2011.

4 A tiered system of local government throughout England has responsibility for services including education, housing and Social Services. For example, across England there are 152 separate Local Education Authorities (LEAs), each of which has responsibility for providing child education in their area. The responsibility for the provision of Social Services and housing will rest with either one of the 152 principal authorities or, particularly in large urban areas, devolved to one of 326 lower tier authorities. Until April 2013 (ie, within the timeframe for the Building Blocks trial), 10 strategic health authorities existed across England, with health care provided through local NHS Primary Care and Hospital Trusts. Subsequent to the trial period and from 1st October 2015 the responsibility for commissioning public health services for children aged 0-5 transferred from NHS England to local authorities
5 Locally available services would exclude universally available services, which may be provided across all sites (whether provided specifically for women of a certain age or all women). Hence, routine midwifery care (for example) would not be reported here.

For peer review only - http://bmjopen.bmj.com/site/about/guidelines.xhtml

c:\programdata\activepdf\dc_ent\tmp\456441\28623087_file000006_653285914.docx

Not all universal services were reported from all sites although these would have been available (eg, universal education provision to age 16 years). In the domains of mental health, addiction and complex needs provision a small number of sites reported no additional locally available or specialist services. No sites reported specialist health visiting services for teenagers. Fourteen sites reported the employment of specialist teenage pregnancy midwives. Details from local informants describe the type and range of services available across the range of providers and sector domains. Services were numerous, complex and in some cases with fluid boundaries facilitating multi-disciplinary interaction to support users. Individual services although provided with similar intent could vary by site, while administrative boundaries between services were shown to be fluid.

Service usage during the trial

Initially 823 women were allocated to receive FNP and 822 women to Usual Care (UC) and following mandatory or elective withdrawal (including of consent), 808 and 810 women respectively completed baseline assessment.[8] The median ages (25th to 75th centile) of women were 17.9 (17.0 to 18.8) in the FNP arm and 17.9 (16.9 to 18.8) in the UC arm. Interviews were completed with 501 women (FNP) and 466 women (UC) at 18 months. At 24 months follow-up the number of interviews completed were 595 (FNP) and 559 (UC). The first woman was recruited to the trial on June 16th 2009 and the date of the last follow-up (24 month) assessment was April 24th 2013.

Community health visiting, midwifery and FNP
Core publicly funded services for mothers are maternity care and health visiting. The mean number of all home visits from health visitors was similar in both study arms (UC: 5.01 (SD 5.51); FNP: 4.70 (SD 7.81)). Contact with health visitors in clinic was quite different with more reported by mothers in the UC arm (mean 6.31, SD 7.07) than in the FNP arm (0.70, SD 2.92). The number of contacts within each reporting period up to 18 months reflects a similar pattern (table 2). The mean number of community midwifery contacts during pregnancy for the 422 UC women responding in late pregnancy was 10.69 (SD: 5.34) and for the 459 in the FNP arm was 10.68 (SD: 5.25). Women allocated to FNP received an average of 9.71, 18.63 and 13.22 valid FN visits per programme phase (Pregnancy, Infancy, Toddlerhood) with average visit duration of 79.14 minutes. There was a programme attrition rate by phase of 3.6%, 10.1% and 7.9% respectively (cumulative rate of 21.1%).

For peer review only

c:\programdata\activepdf\dc_ent\tmp\456441\28623087_file000006_653285914.docx

Table 2 Participant reported access to health services (health visitor and contraception) by follow-up (month)

	6		***12***		***18***		**Combined (up to 18 months)**	
	FNP n=511	*UC n=470*	*FNP n=514*	*UC n=483*	*FNP n=501*	*UC n=466*	*FNP n=501*	*UC n=466*
**Health visitor contacts** *Mean (SD)*								
Home	3.07 (6.08)	3.35 (3.58)	1.24 (3.67)	1.16 (2.63)	0.50 (2.50)	0.93 (2.58)	4.70 (7.81)	5.01 (5.51)
Clinic	0.51 (2.12)	3.72 (5.04)	0.20 (1.37)	1.66 (2.76)	0.06 (0.45)	1.01 (2.51)	0.70 (2.92)	6.31 (7.07)
**Contraceptive services** *%*								
GP surgery	42.3	38.3	41.2	44.1	38.5	46.1		
Family planning clinic	26.2	19.8	19.6	18.6	22.6	18.7		
Children's centre	1.4	0.6	1.0	0.8	1.0	0.4		
Sexual health clinic	6.1	4.5	4.7	4.3	7.2	4.5		

We explored variation in core service usage to determine whether level of observed support (</=3 HV home visits / >3+ HV home visits in the first 6 months postpartum) was directed to participants distinguishable on the basis of baseline characteristics (Table 3). Women who had ever been homeless, had a higher subjectively defined social status, and poorer mental health were associated with 4 or more visits, while visit frequency also varied by trial site (but was not subsequently entered into the final model) (table 3). Homelessness (OR=1.80, 95% CI=1.02 to 3.17) and subjective social status (OR=1.13, 95% CI=1.01 to 1.27) were the only two individual characteristics that remained independently associated with visit numbers.

For peer review only - http://bmjopen.bmj.com/site/about/guidelines.xhtml

c:\programdata\activepdf\dc_ent\tmp\456441\28623087_file000006_653285914.docx

Table 3 Baseline predictors of number of home visits from health visitor by six months postpartum for women in usual care arm

	3 or fewer visits (N=155) N	Median (IQR[a]) or %	4 or more visits (N=312) N	Median (IQR[a]) or %	Overall N=467) Median (IQR[a]) or N (%)	Univariate assoc.[g]
**Age in years**		17.9 (17.1 – 18.7)		17.8 (16.9 – 18.9)	17.8 (16.9 – 18.8)	0.721
**Ethnic background**						0.070
White	130	83.9	276	88.5	406 (86.9)	
Mixed	5	3.2	18	5.9	23 (4.9)	
Asian	3	1.9	4	1.3	7 (1.5)	
Black	15	9.7	13	4.2	28 (6.0)	
Other	2	1.3	1	0.3	3 (0.6)	
**Relationship status**						0.433
Married	4	2.6	3	1.0	7 (1.5)	
Separated	13	8.4	34	10.9	47 (10.1)	
Closely inv. / boyfriend	120	77.4	244	78.2	364 (77.9)	
Just friends	18	11.6	31	9.9	49 (10.5)	
**Live with father of baby**						0.512
Yes	42	27.1	71	22.8	113 (24.2)	
No	108	69.7	212	67.9	320 (68.5)	
Not answered	5	3.2	29	9.3	34 (7.3)	
**Subjective social status:**						
**Family**	155	5.8 (5.0 – 7.0)	309	5.8 (5.0 – 7.0)	5.8 (5.0 – 7.0)	0.896
**Personal**	154	6.8 (5.0 – 8.0)	311	7.1 (6.0 – 8.0)	6.7 (6.0 – 8.0)	**0.007[g]**
**NEET[b]**:	138		266			0.210
Yes	45	32.6	105	39.5	150 (37.1)	
No	93	67.4	161	60.5	254 (62.9)	
**Receive any benefits**	154		311			0.776
Yes	48	31.0	101	32.4	149 (31.9)	
No	106	68.4	210	67.3	316 (67.7)	
Not answered	1	0.6	1	0.3	2 (0.4)	
**Ever been homeless**						**0.023[g]**
Yes	19	12.3	65	20.8	84 (18.0)	
No	136	87.9	247	79.2	383 (82.0)	
**Deprivation (IMDS)[c]**	154	40.4 (24.8 – 54.3)	308	38.0 (24.8 – 51.4)	38.8 (24.8 – 51.7)	0.175
**Health utility**						0.374
Perfect health	104	67.1	195	62.5	299 (64.0)	
Less than perfect health	51	32.9	115	36.9	166 (35.5)	
Not answered	0	0.0	2	0.6	2 (0.4)	
**Self-rated health**						0.227
Excellent	24	15.5	58	18.6	82 (17.6)	
Good	113	72.9	200	64.1	313 (67.0)	
Fair	17	11.0	48	15.4	65 (13.9)	
Poor	1	0.6	6	1.9	7 (1.5)	
**Limiting chronic illness**:						0.144
Yes	24	15.5	66	21.2	90 (19.3)	
No	131	84.5	246	78.8	377 (80.7)	

For peer review only

c:\programdata\activepdf\dc_ent\tmp\456441\28623087_file000006_653285914.docx

**Self-efficacy**[d]	151	29.7 (27.0 – 32.5)	308	29.9 (28.0 – 32.0)	29.8 (27.0 – 32.0)	0.604
**Adaptive functioning:** [e] **Difficulty in at least one basic skill**						0.674
Yes	36	23.2	78	25.0	114 (24.4)	
No	119	76.8	234	75.0	353 (75.6)	
**3 or fewer key life skills**						0.822
Yes	39	25.2	81	26.0	120 (25.7)	
No	116	74.8	229	73.4	345 (73.9)	
Missing	0	0.0	2	0.6	2 (0.4)	
**At least one burden**						0.080
Yes	55	35.5	87	27.9	142 (30.4)	
No	98	63.2	224	71.8	322 (69.0)	
Missing	2	1.3	1	0.3	3 (0.6)	
**Alcohol / drug use**[f]	147	1.2 (0.0 – 2.0)	296	1.3 (0.0 – 2.0)	1.3 (0.0 – 2.0)	0.212
**Antisocial behaviour**	154	2.0 (1.0 – 3.0)	310	2.3 (1.0 – 4.0)	2.2 (1.0 – 3.0)	0.088
**Social support**	155	85.7 (77.0 – 98.7)	310	85.8 (79.0 – 98.7)	85.8 (77.6 – 98.7)	0.491
**Relationship quality**	130	28.5 (26.0 – 32.0)	255	28.2 (26.0 – 32.0)	28.3 (26.0 – 32.0)	0.433
**Family resources**	150	13.5 (11.0 – 16.0)	296	13.5 (11.0 – 16.0)	13.5 (11.0 – 16.0)	0.884
**Psychological distress / Mental health**	155	20.3 (15.0 – 25.0)	311	21.8 (17.0 – 26.0)	21.3 (16.0 – 26.0)	0.025
**Trial site**						0.003[h]
1	1	0.6	10	3.2	11 (2.4)	
2	5	3.2	8	2.6	13 (2.8)	
3	14	9.0	15	4.8	29 (6.2)	
4	2	1.3	7	2.2	9 (1.9)	
5	8	5.2	10	3.2	18 (3.9)	
6	6	3.9	7	2.2	13 (2.8)	
7	7	4.5	7	2.2	14 (3.0)	
8	12	7.7	19	6.1	31 (6.6)	
9	13	8.4	26	8.3	39 (8.4)	
10	5	3.2	17	5.4	22 (4.7)	
11	7	4.5	30	9.6	37 (7.9)	
12	17	11.0	16	5.1	33 (7.1)	
13	7	4.5	35	11.2	42 (9.0)	
14	5	3.2	3	1.0	8 (1.7)	
15	11	7.1	26	8.3	37 (7.9)	
16	19	12.3	19	6.1	38 (8.1)	
17	8	5.2	30	9.6	38 (8.1)	
18	8	5.2	27	8.7	35 (7.5)	

a Interquartile range; b Definition of NEET: Not in education employment or training (applicable only to those whose academic age is >16 at baseline interview); c Higher IMD score indicated more deprivation; d Higher score indicates higher level of self-efficacy; e Higher score indicates better management of day-to-day lives and routines (for each of the three sub-scales); f CRAFFT screening test[11] for substance related risks and problems in adolescents; g italics indicate variable included in logistic regression, bold indicates variable remained significantly associated with number of visits in logistic model; h not modelled in regression analysis due to high number of levels ; i The three original scale items comprised having to care for someone with long-term illness or alcohol / drug problem, feeling that they had in/sufficient privacy, living with people who respondents wished were not around

Other services

For peer review only - http://bmjopen.bmj.com/site/about/guidelines.xhtml

c:\programdata\activepdf\dc_ent\tmp\456441\28623087_file000006_653285914.docx

Participants accessed a wide range of services encompassing housing support, financial support, education, healthcare, childcare and social care (tables 4 to 5). A small proportion of respondents reported accessing support for housing outside of their friends and family, mostly from the local authority (table 4). The small difference in reported rates between study arms would appear to have been in part attributable to additional assistance from the FNP family nurse. Most participants reported being in receipt of additional publicly funded financial support. For most participants this included income support, housing benefit and council tax reductions with similar rates between study arms reported. Smaller proportions of participants reported other forms of financial assistance related to employment, education or personal health (eg, Jobseekers allowance). The largest difference in reported rates between study arms was for those who received regular financial support from parents: 8.9% (FNP), 15.4% (UC).

Table 4 Participants (%) reporting housing and financial support by follow-up point (months)

	**6**		**12**		**18**		**24**	
	FNP n=511	*UC n=470*	*FNP n=514*	*UC n=483*	*FNP n=501*	*UC n=466*	*FNP n=595*	*UC n=559*
i) Source of housing support								
Anyone outside of friends or family	18.0	14.9	12.1	9.9	9.2	8.4	12.1	9.7
Local authority housing department	7.0	6.6	5.1	5.6	4.6	4.7	6.2	5.9
Family Nurse	4.1	-	3.1	-	2.2	-	5.4	-
ii) Source of financial support								
State benefits or payments	-		-		-		86.9	88.4
Income support	-		-		-		62.0	63.3
Jobseekers allowance	-		-		-		8.6	8.9
Housing benefit	-		-		-		64.2	68.5
Council tax reduction	-		-		-		62.9	63.3
Disability living allowance	-		-		-		2.5	5.4
Incapacity benefit	-		-		-		0.7	1.6
Child Support Agency[1]	-		-		-		12.8	11.6
Regular support from parents	-		-		-		8.9	15.4
Education grants	-		-		-		5.5	5.9

1 Directly or via partner

Most women seeking contraception obtained it from their general practice, and to a lesser extent from a family planning clinic. There were some small differences between study arms by time point (eg at 18 months 46.1% of women in the UC arm accessed contraception from their GP, while 38.5% in the FNP did) but overall use of this service was similar. The proportion of women accessing any education gradually increased across the duration of the trial. By 24 months about a fifth of women were in school, college or training (FNP: 22.5%, UC: 18.1%). This was mostly in mainstream education, although there were a small number of women in both trial arms accessing support in more specialised units (eg, learning support unit). A similar pattern of increasing support for childcare was observed over time with approximately a quarter of women reporting some form of childcare support used at 24 months. Support was received from a variety of sources and there appeared to be a similar pattern of usage between study arms.

For peer review only - http://bmjopen.bmj.com/site/about/guidelines.xhtml

c:\programdata\activepdf\dc_ent\tmp\456441\28623087_file000006_653285914.docx

Table 5 Participants (%) reporting access to education, childcare and other support services by follow-up point (months)

	6		12		18		24	
	FNP n=511	*UC n=470*	*FNP n=514*	*UC n=483*	*FNP n=501*	*UC n=466*	*FNP n=595*	*UC n=559*
i) Education attended								
Any school, college or training	14.5	16.4	20.4	19.0	22.4	20.6	22.5	18.1
Mainstream school or college	11.3	13.7	15.0	15.6	19.5	18.7	16.6	12.7
Learning support unit	0.6	0.2	0.6	0.6	0.2	0	0.7	0.7
Pupil referral unit	0	0.2	0	0	0	0	0	0.2
Teenage mums support unit	0.8	1.7	0.6	0.6	0.4	0.6	0.7	1.5
ii) Childcare accessed								
Any childcare	7.0	7.0	16.1	13.3	25.5	21.5	26.9	24.3
Crèche at school or college	4.1	4.5	8.8	6.6	4.8	3.6	12.1	12.3
Day nursery at children's centre	0.8	0.6	0	0	3.6	2.4	5.5	4.3
Child-minder	1.8	1.1	2.1	1.2	3.2	2.4	3.2	3.0
Other forms of childcare	0.8	0.6	2.1	2.9	8.0	6.9	6.7	6.1
iii) Other support services								
Connexions	31.1	26.8	23.5	23.2	16.8	17.0	*	*
School nurse	1.4	1.5	0.8	0.4	0	0.9	0.5	0.9
Young People's Centre	4.9	7.0	2.7	3.9	1.8	1.9	1.8	1.6
Family Information Centre	2.0	2.3	1.2	1.5	2.2	3.0	1.3	1.4
Children's Centre	36.6	36.6	25.8	35.6	28.3	30.0	34.6	26.7
Child development centre	0.6	0.6	0.4	1.7	0.8	1.5	1.0	2.5
Crèche/ day nursery	10.8	10.8	15.4	14.7	8.4	6.0	17.6	16.6
Toddler group	7.8	7.9	12.5	11.0	16.2	15.2	19.2	21.5
Leaving care service	1.4	0.4	1.8	1.0	1.4	0.6	2.0	0.9
Fostering service	0.6	0.2	0.4	0.4	0	0.6	0.3	0.4
Youth offending team	0.8	0.9	0.2	0.2	0.4	0	0.3	0
Social worker	10.6	10.0	7.4	7.5	8.2	6.2	13.1	9.7
Alcohol / drug support	0.6	0	0.2	0.2	0	0.4	0.3	0.5

1 Some respondents indicated they were in school, college or training but provided no further information
** Not collected as service reconfigured*

Various other services were accessed, the most frequent being Connexions and Children's Centres. The former was used with decreasing frequency over time (consistent with the aging profile of the sample), while the latter showed a more variable pattern of access across each time point and on occasions quite different rates of access between trial arms. At six months one in ten mothers in both trial arms reported contact with a social worker, a rate that varied over time to 24 months at which point there was only a small difference between groups (FNP: 13.1%, UC: 9.7%).

DISCUSSION

To understand the service context within which FNP was trialled we mapped the range of services available. The multiplicity of services often within the same area and their varying labels often concealed similarities and differences between services. We established the usage of key services by trial participants across service domains. We particularly focused on those most directly relevant to the intervention (eg, health visiting) although included many other services. With mostly only small differences in usage between trial arms perhaps what is most important is the wide range of services being accessed. Although the previous US

For peer review only

c:\programdata\activepdf\dc_ent\tmp\456441\28623087_file000006_653285914.docx

trials have not further reported on broader services, the contexts were likely to be very different from the English trial setting.

FNP aims to impact upon a range of maternal and child outcomes. Therefore our selection of relevant services was necessarily broad and informed by the intervention's theory of change, which includes promoting access to services. However, previously reported attempts to map services have been challenging even when restricted to a single organisation.[12] To cope with such complexity researchers have sought to distinguish between specialist and generic services, including through a multi-staged approach as used here.[13] It has been consistently reported that information provision is time consuming for professionals (or other key informants) in such exercises, as we also found.[14] Individual informants may be unfamiliar with all relevant services even within their professional area, hence the coordinated approach to data gathering from multiple informants we used. Feedback from FNP staff in our process evaluation focus groups highlighted a similar challenge when acquiring knowledge about local services, essential for then linking up clients to relevant support.[10] Some core services such as mainstream education were not always reported and illustrates the need to clearly define the scope of the information request to informants, especially the boundaries within which they are being asked to respond. On this last point we would also clarify that many services however resourced and whether universal in availability or not, may impact upon the health and wellbeing of mother and child. We have measured for trial participants services actually used. The extent to which mothers can practically access currently unused or underused services effectively represents a key potential for future benefit if addressable barriers to accessed can be removed.

Our experience from this study will encourage us to further develop an approach to better understanding usual care in complex service settings. Our approached spanned an elicitation phase whereby we started by plotting a map of services and then a consolidation phase where we largely sought to confirm the contours on the map. Accordingly we took an exploratory approach for the former and a largely confirmatory approach for the latter. How either is actually done may depend on study setting and resource. The spreadsheets worked well in that they were portable and could be transferred easily to informants for completion once we had piloted them. However, an in-person semi-structured approach could have worked as well, but may have been more resource intensive. The complexity and number of services identified would have been unfeasible to include in their entirety in the trial's participant follow-up survey, but that may be important in some other studies. For example, if it was considered that sites clearly varied in provision of key services, gaining high quality information about such site characteristics could inform more informative analysis such as multi-level modelling. Finally, we initially explored the nature of available services with professionals, and only then asked mothers about services actually used via a mostly structured list of options. An exploratory exercise with mothers may well have shed light on other potential relevant services.

In effectiveness trials existing services could respond by augmenting support to those in the control arm. Such performance bias limits generalizability especially if that support was very different from usual care and approaching the level of support provided by the new intervention. Our findings do not indicate this in general and specifically for community midwifery and health visiting, the two most closely aligned universal services. However determining only the number of contacts may mask enhanced support provided in the form of longer contacts, or contacts from specialist practitioners. Community midwives visits were equivalent between trials arms and the difference in contacts with health visitors was attributable to clinic rather than home visits and therefore unlikely to be substantial. There was some indication that women in the usual care arm with some additional objective need identified at baseline, such as experience of homelessness, received more home visits. However, providing enhanced care to clients most in need would be usual practice. Evidence that this occurred in a trial context is not in itself a threat to external validity. The large caseloads managed by health visitors emphasises the lack of opportunity to provide significant additional support to mothers allocated to usual care.[10]

Our trial found fewer short-term benefits than previous US trials despite FNP being well implemented.[1-3, 8] The population we studied differed from that in the US, for example by

For peer review only - http://bmjopen.bmj.com/site/about/guidelines.xhtml

being fundamentally identified by maternal age and this may have contributed to some differences in impact detected. The upper age limit for women in the US trials was greater in each case than in England, and they also could have been enrolled at a later stage of gestation, for example, before delivery in Denver. In the three US trials the intervention had been provided by a total of 5 (Elmira), 12 (Memphis) and 10 (Denver) nurses in single areas with study samples of 400, 1138 and 735 women respectively. In our trial 131 nurses delivered the intervention across 18 local sites. The English service context would have been very different. Some additional standardised support in the form of developmental screening and referral, and free travel to appointments was provided to women in the control arm of each US trial. However, the broad and layered range of services identified in our study would not have been available. The broader adverse social context present in the first US trial and from which much longer-term evidence has been derived has limited direct comparison. For example, at the inception of the first US trial, Elmira was ranked bottom of all 380 US metropolitan statistical areas in terms of economic conditions.. That is not to say that women in our trial were free of disadvantage or had services that fully met their needs. However, substantial differences across trial settings and the substantial duration between the trials are likely to have varied the potential for beneficial impact.

Service provision may change over time and any single mapping exercise will miss this real-world dynamic. We conducted telephone interviews with five FN supervisors towards the end of the trial. These explored whether there had been any key changes to local service provision. Recent major change was mostly not identified as occurring although the reduction in Connexions services was flagged up. Quantification of service use should be open to the capture of newer services. Additionally, with superficial service names not always reflecting well actual support provided it is important to look beyond service labels. Finally, high-level service descriptions do not always represent the often complex multi-professional interactions which necessarily facilitate service delivery. This emphasises the need for adequate qualitative description and interpretation of services.

Loss to follow-up at assessment points may introduce bias into the descriptive analysis. We have previously reported on group differences in attrition apparent at 24 months follow-up however such, differences were small.[10] A second consideration is the level of detail available for health visitor and midwifery contacts (eg, visit duration). It is reasonable to assume that given capacity and opportunity, women in the UC arm visited by health professionals would have received greater attention than other clients perceived as less in need. This is consistent with their professional role and reflective of contemporary best usual practice.[15] It is also possible that women in the FNP arm received relatively less attention than non-FNP clients if they were seen to be receiving enhanced support. Nevertheless, the total number of home and clinic visits received in both trial arms was small compared to that provided by FNP nurses. Future process evaluations should model the impact upon existing services of such service innovation to both avoid unintended consequences (eg, service displacement) and maximise synergy across services.

Moore and colleagues recommend primarily qualitative methods for capturing unanticipated or complex intervention pathways, which in this instance we take to be impact upon co-existing services.[17] They also emphasised the need to capture the mechanisms using logic models including where these reflected broader context. The extent to which an intervention's impact could actually induce harm either at the individual level or within a system can further be reflected by use of a dark logic model.[18] Bonell and colleagues recommend approaches to developing such a logic model, for example, by hypothesising how the agency of key stakeholders may interact with social structures to produce unintended consequences. Reflection in such model building could be informed by the use of mid-range sociological or psychological theory. This could also be combined with exploratory qualitative work with local stakeholders (eg service managers or practitioners) well placed to observe both intended and unintended intervention impacts. This is also consistent with approaches which recognise the implementation of public health interventions occurring within complex adaptive social systems, such as May's Normalisation Process Theory.[19] NPT identifies implementation as occurring in a dynamic, non-linear and emergent fashion. This offers a broader theoretical context within which to explore not only how one intervention becomes adapted to its

For peer review only - http://bmjopen.bmj.com/site/about/guidelines.xhtml

c:\programdata\activepdf\dc_ent\tmp\456441\28623087_file000006_653285914.docx

environment and may vary but also how that social context and usual services may also become adapted too.

The effectiveness of a public health intervention can only be adequately evaluated with a sound understanding of the service context within which it operates and which may also form the trial comparator.[16] Describing and quantifying the nature of usually available services can be challenging especially when services arise from a number of sectors, may evolve over the period of study and vary across study sites. In mapping the pattern of support potentially available to participants in our trial we have gained a critical understanding of the context within which and against which FNP should be considered. In quantifying maternal reported service usage we have provided key insights into how our main trial results should therefore be interpreted. While challenging, we remain convinced of the need to develop this area of research when evaluating public health interventions. Indeed, in their feedback survey respondents reported the usefulness of the exercise in gaining greater insights about local services, some sharing the generated service summaries with their teams.

For peer review only

c:\programdata\activepdf\dc_ent\tmp\456441\28623087_file000006_653285914.docx

ACKNOWLEDGEMENTS
The authors gratefully acknowledge the contribution of Dr Lesley Wye, University of Bristol and Dr Marie-Jet Bekkers who contributed to some of the planning and implementation of the reported study, and to all other members of the broader Building Blocks research team who supported overall trial delivery. We are grateful to each local site informant who contributed to data collection in either of the two rounds of service mapping and especially to the three local coordinators who initially piloted our mapping tool. We are also grateful to all women who participated in the trial and for their time in completing outcome and resource assessments.

COMPETING INTERESTS
Prior to working on the Building Blocks trial, Sue Channon facilitated two workshops on Motivational Interviewing for supervisors within the FNP. The authors declare no competing interests.

CONTRIBUTORS
MR, KH and JS conceived the study and all authors contributed to the development of its protocol. MR wrote the first draft with further contributions from all authors. RP, JS and GM were involved in data collection and management. RP was responsible for developing the survey of local stakeholders and GM was responsible for managing data collected from trial participants used in the analysis. RP, RCJ, GM and MR were involved in analysis and developing summary tables for publication. SC and JS were responsible for the management of this package of work within the trial overall. MR was responsible for obtaining study funding. All authors contributed to data interpretation, reviewed successive drafts and approved the final version of the manuscript.

ETHICS
The trial was approved by the Wales NHS Research Ethics Committee (09/MRE09/08) and received governance approval from all participating NHS sites. All women provided written informed consent.

PARTICIPANT CONSENT
All participants provided written informed consent to take part in the study.

FUNDING
The Centre for Trials Research, Cardiff University is funded by the Welsh Government through Health and Care Research Wales and the authors gratefully acknowledge the Centre's contribution to study implementation. This is an independent report commissioned, sponsored and funded by the Policy Research Programme in the Department of Health (reference no. 006/0060). The views expressed are not necessarily those of the Department of Health.

DATA SHARING
The datasets generated and analysed during the current study are not publicly available as contributors / participants may be identifiable and are also subject to sponsor approval, but may be available from the corresponding author on reasonable request.

For peer review only - http://bmjopen.bmj.com/site/about/guidelines.xhtml

c:\programdata\activepdf\dc_ent\tmp\456441\28623087_file000006_653285914.docx

References

1. Olds DL, Henderson CR, Jr., Chamberlin R, Tatelbaum R. Preventing child abuse and neglect: a randomized trial of nurse home visitation. Pediatrics 1986; 78 (1): 65-78.
2. Kitzman H, Olds DL, Henderson CR, Jr., et al. Effect of Prenatal and Infancy Home Visitation by Nurses On Pregnacy Outcomes, Childhood Injuries, and Repeated Childbearing: A Randomised Controlled Trial. JAMA 1997; 278 (8): 644-52.
3. Olds DL, Robinson J, O'Brien R, et al. Home Visiting by Paraprofessionals and by Nurses: A Randomized, Controlled Trial. Pediatrics 2002; 110 (3): 486-96.
4. Barnes J, Ball M, Meadows P et al. Nurse-Family Partnership Programme: First Year Pilot Sites Implementation in England - Pregnancy and the Post-partum Period. London: Birkbeck, University of London; 2008.
5. Barnes J, Ball M, Meadows P et al. Nurse-Family Partnership Programme: Second Year Pilot Sites Implementation in England - The Infancy Period. London: Birkbeck, University of London; 2009.
6. Barnes J, Ball M, Meadows P, et al. The Family-Nurse Partnership Programme in England: Wave 1 implementation in toddlerhood & a comparison between Waves 1 and 2a of implementation in pregnancy and infancy. London: Institute for the Study of Children, Families and Social Issues: Birkbeck, University of London, 2011.
7. Owen-Jones E, Bekkers M-J, Butler CC, et al. The effectiveness and cost-effectiveness of the Family Nurse Partnership home visiting programme for first time teenage mothers in England: a protocol for the Building Blocks randomised controlled trial. BMC Pediatrics 2013; 13 (1): 114.
8. Robling M, Bekkers M-J, Bell K, et al. Effectiveness of a nurse-led intensive home-visitation programme for first-time teenage mothers (Building Blocks): a pragmatic randomised controlled trial. Lancet 2015; published online Oct 14. http://dx.doi.org/10.1016/S0140-6736(15)00392-X.
9. NVivo qualitative data analysis Software; QSR International Pty Ltd. Version 8, 2008
10. Robling MR Bekkers M-J, Bell K et al. Evaluating the Family Nurse Partnership Programme in England: A Randomised Controlled Trial. Final report to The Department of Health. 2015. (http://bit.ly/buildingblockstrial).
11. Knight JR, Sherritt L, Shrier LA et al Validity of the CRAFFT substance abuse screening test among adolescent clinic patients. Arch Pediatr Adolesc Med 2002, 156(6):607–614.[CRAFFT].
12. Hotopf M, Rayner L, Valsraj K. Improving 'Whole Person Care' A report on mental health related services offered by King's Health Partners' acute trusts: King's Health Partners, 2012.
13. Challis D, Darton R, Donnelly M et al. Mapping and evaluation of care management arrangements for older people and those with menatal health problems in an integrated health and social services system: a comparative study: The Personal Social Services Research Unit, 1999.
14. Health Do. Children's services mapping. A complete picture of children's service provision: Department of Health. Department for Children Schools and Families, 2010.
15. NICE. Pregnancy and complex social factors: A model for service provision for pregnant women with complex social factors. London, 2010.
16. Smelt AFH, van der Weele GM, Blom JW et al. How usual is usual care in pragmatic intervention studies in primary care? An overview of recent trials. British Journal of General Practice 2010 e305.
17. Moore GF, Audrey S, Barker M, Bond L, Bonell C, Hardeman W, Moore L, O'Cathain A, Tinati T, Wight D, Baird J. Process evaluation of complex interventions: Medical Research Council guidance. BMJ 2015;350:h1258 doi:10.1136/bmj.h1258
18. Bonell C, Jamal F, Melendez-Torres GJ, Cummins S. 'Dark logic': theorising the harmful consequences of public health interventions. Epidemiol Community Health 2015;69:95–98.
19. May CR, Johnson M, Finch T. Implementation, context and complexity. Implementation Science (2016) 11:141

For peer review only - http://bmjopen.bmj.com/site/about/guidelines.xhtml

Appendix 1

Example of service mapping content / user instructions from 'Education' worksheet

Please describe services available for teenage, first-time mothers in your area	**Name of service (If applicable)**	**Description of service / Care**	**Are there any limits on the availability of this service? E.g., Number of women that can be offered the service**
Is there a Mother and Baby hostel in your area?	Mother and baby hostels	Dedicated hostels for homeless women who are pregnant or have a new baby. Additional support both from trained staff	Some will take young women in the early stages of pregnancy (up to 6 months approx). Some will not house pregnant women, or young babies
What temporary accommodation can be offered?	Hostels	Temporary accommodation with varying degrees of support, usually containing some shared facilities	Most require assessment of eligibility and suitability. Not usually suitable for those under 18, or youths with offending behaviour.
Temporary accommodation (for the temporary homeless)?	Emergency hostels and night shelters (or bed and breakfasts if nothing else is available - usu max of 6 weeks)	Offer somewhere to sleep, food, warmth and hygiene. Residents are normally asked to pay a small additional contribution for their meals.	Usually a direct access/first-come-first served basis. Night shelters are usually free. Most hostels charge.
Charity involvement?	LIFE Housing	Provides a support service (General Support, Individual Support Plans and the LIFE Skills Programme) and community outreach schemes	(Not specified)
Foyers	Foyers	Integration of accommodation and support services: training in basic/independent living skills, inc. ongoing support when the young person has left the Foyer	Most foyers have a waiting list. Some foyers only accept referrals from local councils
Women's refuges	Women's refuges	A refuge is a safe house where women and children who are experiencing domestic violence can stay free from abuse	Refuges are highly unlikely to accept women from their immediate local area. Some are for women w/ part' ethnic/cultural backgrounds
Housing Associations / RSLs (Registered Social Landlords)	Housing associations / RSLs (Registered Social Landlords)	Provide homes for people on low incomes. Some housing associations specialise in accommodation for particular groups of people, such as younger people	Long waiting list. Chance of place/waiting time depends on personal circumstances (e.g. children), and other factors
Supported lodgings schemes	Supported lodgings	Individuals in the community offer a room in their home with varying degrees of support. A safe and supportive environment for young people	Suitable for youths: leaving care, deemed vulnerable and in need, requiring temp acc, or with no statutory entitlement to housing

For peer review only

Supported housing	Self-contained, cluster and shared arrangements with varying degrees of support	Supported housing will usually provide housing-related support to help a young person prepare for independent living	Many schemes accessible via social services' leaving care arrangements, and via housing departments for young homeless people
Mainstream housing	Independent accommodation	Independent accommodation. Housing & social services may have arrangements w/ private landlords to provide accommodation for vulnerable youths	Housing benefit restrictions apply to under 25s living in the private sector and applicants are limited to the single-room rent housing benefit level
Nightstop schemes	Emergency accommodation (Depaul Nightstop UK)	Nightstop schemes provide emergency accommodation for young homeless people aged 16–25 in the homes of a network of volunteer hosts	Referrals to service after risk assessments by a recognised agency; such as social services, Connexions, police, housing depts
Supported housing	Supported housing schemes	Housing schemes offering accommodation linked with on-site or outreach support from dedicated staff (practical and emotional help)	Schemes vary in size
Custodial institution	Secure Children's Homes	Concentrate on physical, emotional, behavioural needs, & aim to give youths individually tailored support to resolve the issues that led them to commit an offence	These are relatively small institutions, with between 6 and 40 beds and a high staff to young person ratio
Floating support	Floating support services	To sustain a tenancy through the dev' of independent living skills. Provides general, non-specialist support with daily living skills, practical tasks or emotional support which promotes or maintains a person's ability to live in their own home	Reviews of the progress and support plan ensure that the services adjust appropriately to changing needs. An exit strategy determines when support is withdrawn. If a person needs support later, it can return to them

For peer review only

Building Blocks Service Mapping Report

STROBE Statement—checklist of items that should be included in reports of observational[1] studies

	Item No	Recommendation	Page
**Title and abstract**	1	(*a*) Indicate the study's design with a commonly used term in the title or the abstract	1
		(*b*) Provide in the abstract an informative and balanced summary of what was done and what was found	2
**Introduction**			
Background/rationale	2	Explain the scientific background and rationale for the investigation being reported	3
Objectives	3	State specific objectives, including any prespecified hypotheses	3
**Methods**			
Study design	4	Present key elements of study design early in the paper	3, 4
Setting	5	Describe the setting, locations, and relevant dates, including periods of recruitment, exposure, follow-up, and data collection	4, 5, 6, 9
Participants	6	(*a*) *Cohort study*—Give the eligibility criteria, and the sources and methods of selection of participants. Describe methods of follow-up<br>*Case-control study*—Give the eligibility criteria, and the sources and methods of case ascertainment and control selection. Give the rationale for the choice of cases and controls<br>*Cross-sectional study*—Give the eligibility criteria, and the sources and methods of selection of participants	5
		(*b*) *Cohort study*—For matched studies, give matching criteria and number of exposed and unexposed<br>*Case-control study*—For matched studies, give matching criteria and the number of controls per case	NA
Variables	7	Clearly define all outcomes, exposures, predictors, potential confounders, and effect modifiers. Give diagnostic criteria, if applicable	10-14
Data sources/ measurement	8*	For each variable of interest, give sources of data and details of methods of assessment (measurement). Describe comparability of assessment methods if there is more than one group	*10-14*
Bias	9	Describe any efforts to address potential sources of bias	NA
Study size	10	Explain how the study size was arrived at	4
Quantitative variables	11	Explain how quantitative variables were handled in the analyses. If applicable, describe which groupings were chosen and why	5
Statistical methods	12	(*a*) Describe all statistical methods, including those used to control for confounding	5
		(*b*) Describe any methods used to examine subgroups and interactions	NA
		(*c*) Explain how missing data were addressed	9*
		(*d*) *Cohort study*—If applicable, explain how loss to follow-up was addressed<br>*Case-control study*—If applicable, explain how matching of cases and controls was addressed<br>*Cross-sectional study*—If applicable, describe analytical methods taking	NA

For peer review only - http://bmjopen.bmj.com/site/about/guidelines.xhtml
For peer review only

	account of sampling strategy	
	(*e*) Describe any sensitivity analyses	NA

**Results**			
Participants	13*	(a) Report numbers of individuals at each stage of study—eg numbers potentially eligible, examined for eligibility, confirmed eligible, included in the study, completing follow-up, and analysed	9*
		(b) Give reasons for non-participation at each stage	9*
		(c) Consider use of a flow diagram	9*
Descriptive data	14*	(a) Give characteristics of study participants (eg demographic, clinical, social) and information on exposures and potential confounders	9*
		(b) Indicate number of participants with missing data for each variable of interest	10-14
		(c) *Cohort study*—Summarise follow-up time (eg, average and total amount)	10-14
Outcome data	15*	*Cohort study*—Report numbers of outcome events or summary measures over time	*10-14*
		Case-control study—Report numbers in each exposure category, or summary measures of exposure	-
		Cross-sectional study—Report numbers of outcome events or summary measures	-
Main results	16	(*a*) Give unadjusted estimates and, if applicable, confounder-adjusted estimates and their precision (eg, 95% confidence interval). Make clear which confounders were adjusted for and why they were included	10-14
		(*b*) Report category boundaries when continuous variables were categorized	NA
		(*c*) If relevant, consider translating estimates of relative risk into absolute risk for a meaningful time period	NA
Other analyses	17	Report other analyses done—eg analyses of subgroups and interactions, and sensitivity analyses	NA
**Discussion**			
Key results	18	Summarise key results with reference to study objectives	14,15
Limitations	19	Discuss limitations of the study, taking into account sources of potential bias or imprecision. Discuss both direction and magnitude of any potential bias	15,16
Interpretation	20	Give a cautious overall interpretation of results considering objectives, limitations, multiplicity of analyses, results from similar studies, and other relevant evidence	14, 15, 17
Generalisability	21	Discuss the generalisability (external validity) of the study results	16
**Other information**			
Funding	22	Give the source of funding and the role of the funders for the present study and, if applicable, for the original study on which the present article is based	18

1 The paper reports an analysis from the process evaluation (including descriptive analysis of service usage data) from a cohort of women participating in a randomised control trial

** Citation to full main trial report included in text*

For peer review only

BMJ Open

What is usual care for teenagers expecting their first child in England?: a process evaluation using key informant mapping and participant survey as part of the Building Blocks randomised controlled trial of specialist home visiting.

Journal:	*BMJ Open*
Manuscript ID	bmjopen-2017-020152.R2
Article Type:	Research
Date Submitted by the Author:	16-Feb-2018
Complete List of Authors:	Robling, Michael; Cardiff University, Centre for Trials Research<br>Cannings John, Rebeca; Cardiff University, Centre for Trials Research<br>Channon, Sue; Cardiff University, Centre for Trials Research<br>Hood, Kerenza; Cardiff University, South East Wales Trials Unit, Centre for Trials Research<br>Moody, Gwenllian; Cardiff University, Centre for Trials Research<br>Poole, Ria; Cardiff University, Medicine<br>Sanders, Julia; School of Healthcare Sciences, Room 12:15, Eastgate House, 35-43 Newport Road
**Primary Subject Heading**:	Public health
Secondary Subject Heading:	Public health, Qualitative research, Research methods
Keywords:	Organisation of health services < HEALTH SERVICES ADMINISTRATION & MANAGEMENT, PAEDIATRICS, Child protection < PAEDIATRICS, PUBLIC HEALTH

SCHOLARONE™
Manuscripts

Title: What is usual care for teenagers expecting their first child in England?: a process evaluation using key informant mapping and participant survey as part of the Building Blocks randomised controlled trial of specialist home visiting.

Corresponding author:

Prof Mike Robling
Centre for Trials Research, Cardiff University
Neuadd Meirionnydd, Heath Park, Cardiff. CF14 4YS
roblingmr@cardiff.ac.uk
+44 (0)29 2068 7177

Authors:

- Mike Robling, Centre for Trials Research, Cardiff University, Cardiff, Wales, UK
- Rebecca Cannings-John, Centre for Trials Research, Cardiff University, Cardiff, Wales, UK
- Sue Channon, Centre for Trials Research, Cardiff University, Cardiff, Wales, UK
- Kerry Hood, Centre for Trials Research, Cardiff University, Cardiff, Wales, UK
- Gwen Moody, Centre for Trials Research, Cardiff University, Cardiff, Wales, UK
- Ria Poole, School of Medicine, Cardiff University, Cardiff, Wales, UK
- Julia Sanders, School of Healthcare Sciences, Cardiff University, Cardiff, Wales, UK

Key words: Public health, child health, nursing, randomised trial

Current abstract word count: 299
Current manuscript word count: 4080
Reference count: 19

For peer review only

Title: What is usual care for teenagers expecting their first child in England?: a process evaluation using key informant mapping and participant survey as part of the Building Blocks randomised controlled trial of specialist home visiting.

Abstract

Objectives

We compared the US-derived Family Nurse Partnership (FNP) home visiting programme when added to usually provided health and social care for first-time teenage mothers, to usual care alone. We aimed to: establish the nature of usual care, measure service usage and assess performance bias in core usual care services.

Design

Within trial process evaluation. Local professionals completed a survey mapping local health and social care services in seven domains. This focused on services available to young women, especially those relevant to pregnant teenagers. Descriptive data were assessed thematically to establish the range of services. Quantitative data collection with FNP supervisors enumerated service provision by site. Services identified were included in main participant trial follow-up interviews at four time-points to quantify usage. Usage was described descriptively by domain. We explored predictors of health visitor visits.

Setting

Eighteen partnerships of local authority and healthcare organisations in England.

Outcomes

Descriptive framework of services. Rates of service usage reported by trial participants.

Results

161 separate services were identified, with multiple service models in each domain, broadly categorised as universal or specialist (eg for teenage mothers). FNP supervisors identified 30-63 universal services per site and 22-67 specialist services. Use of core maternity care services were similar across trial arms and with only small differences in use of health visiting services. Participants accessed a wide range of services. Women who had ever been homeless, who had a higher subjectively defined social status, and poorer mental health received more visits from a health visitor.

Conclusions

The large number of services available to teenage mothers in England may limit the incremental benefit achievable through enhanced home visiting. There was little evidence of compensatory practice, such as additional care for women in the usual care arm. Measuring usual care when trialling complex interventions is challenging and essential.

For peer review only - http://bmjopen.bmj.com/site/about/guidelines.xhtml

Strengths and limitations of this study

- The identification of sometimes multiple local stakeholders and drawing upon their existing knowledge using a semi-structured self-completion tool about a range of relevant services enabled us to develop a rich picture of what may be usually available care for teenagers expecting their first child.
- Undertaking the initial mapping exercise enabled us to develop a more informed service use inventory with greater content validity than may otherwise have been possible.
- The combination of professionally-led key informant mapping and detailed service use recording as part of trial follow-up data collection therefore provides a more nuanced understanding of usual care. This greater understanding of the trial's control condition enhances interpretation of trial results.
- However, changes over time, and within and between site differences in how services are configured, perceived and understood means that a summary statement about all locally relevant services will need to be intermittently revisited.
- Although we have an understanding about how services were similarly or differently accessed by intervention and control participants in the trial, the intensity and duration of individual sessions for non-FNP services is not known. However, comprehensively attempting to collect such detailed data from trial participants would probably not be feasible in practice

For peer review only

For peer review only - http://bmjopen.bmj.com/site/about/guidelines.xhtml

INTRODUCTION

Individual, social, and economic circumstances faced by teenage mothers can challenge a successful start for their children. Responding in 2006, the Government in England adopted a preventative US-derived programme of nurse-led intensive home-visiting, the Family Nurse Partnership (FNP). Specially trained family nurses support first-time mothers through up to 64 home visits starting in early pregnancy and until the child reaches their second birthday. In three US trials, the programme has been evaluated with differing socio-demographic populations, justifying initial testing in a UK context.[1-3]

Following an implementation evaluation, 18 English Primary Care Trust (PCT) sites participated in the Building Blocks trial (ISRCTN23019866) of the programme's effectiveness recruiting 1645 teenagers expecting their first child.[4-8] The sites were dispersed across the UK, and covered two rural and 16 city areas. Women were recruited before 25 weeks gestation, lived within geographical areas served by the FNP team and spoke at least conversational English. Assessing over 60 short-term outcomes (to 24 months post-partum) in domain areas of pregnancy and birth, child development, and maternal life-course, four primary outcomes of programme and policy interest were prioritised.

We compared FNP when added to usually provided health and social care to usual care alone. In the absence of comprehensive public healthcare in the US, across all three previous evaluations the counterfactual was reported as obstetric office-based antenatal care, paediatric developmental screening, referral at specified time points and free transport to office-based consultations. Elevating the control condition to just more than simply no care, the augmented control condition was not further described. Given the provision of free universal health services in the UK, the ethical trial comparator was an active control condition. However, it was expected that what would be available to young families may be complex and vary by site and over time.

We aimed to map and quantify usually provided care and so clarify the trial's control condition, the service context into which FNP was introduced and allow exploration of any performance bias affecting validity of the trial comparison.

METHODS

We first elicited and mapped usual services available locally at each of the 18 trial sites. Each site comprised collaborative partnerships between National Health Service (NHS) organisations and local authorities. All sites had applied to the Department of Heath to be a provider of FNP including by demonstrating local clinical need and commitment to sustain local programme delivery. Sites included urban and rural settings across England and encompassed each of the ten strategic health authorities in England. Second, we enumerated services accessed by participants in both trial arms.

Eliciting and mapping services

A mapping tool was drafted using an Excel worksheet following discussion within the research team. This sought to identify services available for pregnant teenagers and young families across seven initial domains: midwifery, health visiting (specialist public health nurses), education, housing, social care and other services (e.g., Children's Centres) and funding schemes specifically for young parents). This would therefore include services that were also universally available, such as maternity care. The tool required the site contacts to provide the title of service and a brief description. It was piloted with local coordinators at three sites who described service characteristics (e.g., provider, eligibility criteria) and were debriefed by telephone interview to assess feasibility. An amended version, which incorporated completion instructions (Appendix 1), a worked example and study information, was circulated via email in the first instance to each site principal investigator (e.g., the local FNP project lead and in all cases not a member of the research team) who then cascaded to local contacts across health and social care (usually managers or heads of services). By engaging with heads of services and other local professional staff (e.g. housing support workers) further detail about specific services or domains were provided, including

For peer review only - http://bmjopen.bmj.com/site/about/guidelines.xhtml

documentation on local services where available. Respondents were asked to provide details of "routinely provided services within their local authority which may be provided to young women, but may be especially relevant to pregnant teenagers". In parallel with obtaining information describing available services, national policies and guidelines were sourced informing on the minimum expected standard of universally available services such as maternity care and state welfare (e.g. childcare vouchers). Mapping data were collected over six months.

Within sites and across respondents we reviewed submitted returns to identify missing or incomplete data (i.e., to identify the presence or absence of expected services / service descriptions) and followed up if necessary with local site contacts. This process was informed by documentary data provided by sites or available online. Data provided by sites were entered into NVivo 8[9] and analysed thematically by researchers who also involved service experts to review the developing coding framework before coming to a consensus on the final range of services available. A second round of online data collection addressing the same domains aimed to consolidate and confirm information already provided and to reduce variation that may be solely attributable to reporting bias. This comprised a structured form listing services by domain and tick boxes for respondents to indicate presence or absence. Free text ('Other') services allowed for unlisted services to be reported. Local FNP supervisors completed this form.

Enumerating service by trial participants
Trial participants were teenagers (aged 19 years or under at last menstrual period) expecting their first child, living in the catchment area for local FNP provision recruited before 25 weeks gestation, able to provide informed consent and competent to converse in English.[7] Access to supportive services within each core domain was measured as part of the trial's follow-up outcome evaluation telephone interview schedule at late pregnancy, and 6, 12 and 18 months postpartum.[7] These included use of childcare, primary (eg, midwifery, GP, health visiting) and secondary (eg, A&E, Out-patient, In-patients) healthcare attendances, sexual health (contraceptive services), formal education, Connexions (a government funded support and advisory service for young people aged 13-19 years old), support with housing, and a range of additional support services. At 24 months additional questions asked about financial support.

Some data informed the separately reported cost-effectiveness analysis.[10] In the current analysis we describe the pattern of core service usage (eg, health visiting, midwifery, housing) for those in both trial arms, and the level of support provided additionally via FNP (for FNP clients, the Healthy Child Programme was delivered by FNs rather than by health visitors). Data on the latter were provided via the FNP national unit's Information System. Use of services was analysed descriptively and is reported by service domain showing counts and proportions for those in the two trial arms separately. Multivariable logistic regression was used to explore whether certain maternal characteristics collected as part of the trial's baseline assessment were associated with level of observed HV support. We created a binary variable of number of HV visits which distinguished between a standard / expected level of care (less than 4 visits) and enhanced care (4 or more visits). Univariable association were screened using a $p<0.10$ cut off and retained in the final multivariable model. Estimates are shown as odds ratios (ORs) and 95% confidence intervals (CIs).

RESULTS

Eliciting and mapping services

Round one was conducted in a six week period from August 2009. All sites responded, with at least six individual informants contributing data per site. A varying level of detail was provided about identified services. In general spreadsheets circulated to multiple stakeholders were more comprehensively completed.

Similar services within any one domain were subsequently grouped together even if labelled differently by informants. This resulted in 161 identified services, some with similar aims. An example was that of education provided to pregnant teenagers aged under 16 years old with

For peer review only - http://bmjopen.bmj.com/site/about/guidelines.xhtml

eight different named services. In round two, conducted in July 2011, the 161 services were listed, categorised into 12 service domains (the original domains plus ‘other services’ sub-divided on the basis of stage 1 responses into childcare, complex needs, Connexions, drug and alcohol, mental health, third sector, and sexual health).

The total number of services identified per site ranged from 52 to 113. These included between 26 and 53 universal services and between 22 and 86 locally available / specialist services. Services were provided by public, private and third sector organisations and collectively delivered direct care, support or guidance. Examples of Specialist and Locally available services for pregnant teenagers or younger parents are shown in table 1.

For peer review only

For peer review only - http://bmjopen.bmj.com/site/about/guidelines.xhtml

Table 1: Service mapping – examples of services[1] described by study sites[3]

Domain	Specialist services - specifically for pregnant teenagers or younger parents	Locally Available services - with a specialist nature and eligibility criteria, but not necessarily designed for teenage parents
**Education**	• Schools / colleges with provision for teenage mums<br>• Teenage pregnancy support services<br>• Accredited courses with free child care for under 25s	• Home learning programmes
**Housing**	• Teenage parents' scheme: training in independent living skills<br>• Supported housing: young vulnerable women or teenage parents	• Outreach support service aimed at young homeless people under 18<br>• Mother and Baby Hostel
**Health Visiting**		• Antenatal contact at home or in midwife-led antenatal clinics<br>• Minor ailments sessions run by health visitors
**Midwifery**	• Teenage pregnancy midwives<br>• Antenatal clinics run by midwives in schools	• Midwives based in Children's Centres
**Social Services**	• Teenage pregnancy support service	• Targeted youth support for vulnerable young people<br>• Specialist therapeutic unit for young victims of sexual abuse<br>• Family resource service; practical support to access universal services
**Connexions Services[2]**	• Teenage Pregnancy Advisors help young mums-to-be and young families	• Provide information and guidance to Looked After young people<br>• Provide support and guidance for young people leaving care<br>• Provide practical help and advice for young mums who want to go back to college
**Drugs, Alcohol and Smoking**		• Specialist drugs and alcohol services working with police<br>• Community-based young people's drugs and alcohol service<br>• Smoking in Pregnancy cessation service
**Sexual Health**	• Lifestyle services working with teenage parents to prevent second pregnancy	• Family planning services for under 25-yr-olds in community settings<br>• Sexual health services for teenagers<br>• Condom distribution scheme in community settings

For peer review only - http://bmjopen.bmj.com/site/about/guidelines.xhtml

**Mental Health services**		• Specialist Children's and Adolescent Mental Health Services for eating disorders<br>• Mother-and-baby units in hospitals and prisons<br>• Specialist psychiatric unit for postnatal mental illness
**Complex Needs services**	• Support and advocacy for (pregnant) teenagers with complex needs	• Child development centre for pre-school children with complex needs<br>• Sure Start language therapy team<br>• Vulnerable baby service: targeted safeguarding prevention
**Childcare provision**		• Private, voluntary, independent childcare providers<br>• Internet database on county-wide childcare provision
**Local / third sector projects**	• Charity funded teen parents projects<br>• Peer support sessions for teenage fathers-to-be	• Barnardo's Priory Family Centre<br>• Charity funded young parents projects<br>• Home Start: trained volunteers visit mums for approx. 15 months

1 Set information provided by local informants for each reported service included: Name of service, Narrative description, Limits on availability (eg, upper limit on number of women offered service, Location (eg, base), Level of service provision per client (eg, frequency, duration, quantity), Illustrative current caseload, Delivery setting, Client eligibility criteria, Service provider (eg, local authority), Assessment of local service variations compared to other locations
2 A government funded advisory and support service for young people aged 13-19 years old, now discontinued
3 Data collection timing: Round 1 - Data collection was requested over a six-week period from August 2009 to coincide with early stages of trial recruitment; *Round 2:* The survey link was sent to local FNP supervisors for completion in July 2011.

4 A tiered system of local government throughout England has responsibility for services including education, housing and Social Services. For example, across England there are 152 separate Local Education Authorities (LEAs), each of which has responsibility for providing child education in their area. The responsibility for the provision of Social Services and housing will rest with either one of the 152 principal authorities or, particularly in large urban areas, devolved to one of 326 lower tier authorities. Until April 2013 (ie, within the timeframe for the Building Blocks trial), 10 strategic health authorities existed across England, with health care provided through local NHS Primary Care and Hospital Trusts. Subsequent to the trial period and from 1st October 2015 the responsibility for commissioning public health services for children aged 0-5 transferred from NHS England to local authorities
5 Locally available services would exclude universally available services, which may be provided across all sites (whether provided specifically for women of a certain age or all women). Hence, routine midwifery care (for example) would not be reported here.

For peer review only - http://bmjopen.bmj.com/site/about/guidelines.xhtml

Not all universal services were reported from all sites although these would have been available (eg, universal education provision to age 16 years). In the domains of mental health, addiction and complex needs provision a small number of sites reported no additional locally available or specialist services. No sites reported specialist health visiting services for teenagers. Fourteen sites reported the employment of specialist teenage pregnancy midwives. Details from local informants describe the type and range of services available across the range of providers and sector domains. Services were numerous, complex and in some cases with fluid boundaries facilitating multi-disciplinary interaction to support users. Individual services although provided with similar intent could vary by site, while administrative boundaries between services were shown to be fluid.

Service usage during the trial

Initially 823 women were allocated to receive FNP and 822 women to Usual Care (UC) and following mandatory or elective withdrawal (including of consent), 808 and 810 women respectively completed baseline assessment.[8] The median ages (25th to 75th centile) of women were 17.9 (17.0 to 18.8) in the FNP arm and 17.9 (16.9 to 18.8) in the UC arm. Interviews were completed with 501 women (FNP) and 466 women (UC) at 18 months. At 24 months follow-up the number of interviews completed were 595 (FNP) and 559 (UC). The first woman was recruited to the trial on June 16th 2009 and the date of the last follow-up (24 month) assessment was April 24th 2013.

Community health visiting, midwifery and FNP

Core publicly funded services for mothers are maternity care and health visiting. The mean number of all home visits from health visitors was similar in both study arms (UC: 5.01 (SD 5.51); FNP: 4.70 (SD 7.81)). Contact with health visitors in clinic was quite different with more reported by mothers in the UC arm (mean 6.31, SD 7.07) than in the FNP arm (0.70, SD 2.92). The number of contacts within each reporting period up to 18 months reflects a similar pattern (table 2). The mean number of community midwifery contacts during pregnancy for the 422 UC women responding in late pregnancy was 10.69 (SD: 5.34) and for the 459 in the FNP arm was 10.68 (SD: 5.25). Women allocated to FNP received an average of 9.71, 18.63 and 13.22 valid FN visits per programme phase (Pregnancy, Infancy, Toddlerhood) with average visit duration of 79.14 minutes. There was a programme attrition rate by phase of 3.6%, 10.1% and 7.9% respectively (cumulative rate of 21.1%).

For peer review only - http://bmjopen.bmj.com/site/about/guidelines.xhtml

Table 2 Participant reported access to health services (health visitor and contraception) by follow-up (month)

	6		*12*		*18*		**Combined (up to 18 months)**	
	FNP n=511	*UC n=470*	*FNP n=514*	*UC n=483*	*FNP n=501*	*UC n=466*	*FNP n=501*	*UC n=466*
**Health visitor contacts** *Mean (SD)*								
Home	3.07 (6.08)	3.35 (3.58)	1.24 (3.67)	1.16 (2.63)	0.50 (2.50)	0.93 (2.58)	4.70 (7.81)	5.01 (5.51)
Clinic	0.51 (2.12)	3.72 (5.04)	0.20 (1.37)	1.66 (2.76)	0.06 (0.45)	1.01 (2.51)	0.70 (2.92)	6.31 (7.07)
**Contraceptive services** *%*								
GP surgery	42.3	38.3	41.2	44.1	38.5	46.1		
Family planning clinic	26.2	19.8	19.6	18.6	22.6	18.7		
Children's centre	1.4	0.6	1.0	0.8	1.0	0.4		
Sexual health clinic	6.1	4.5	4.7	4.3	7.2	4.5		

We explored variation in core service usage to determine whether level of observed support (</=3 HV home visits / >3+ HV home visits in the first 6 months postpartum) was directed to participants distinguishable on the basis of baseline characteristics (Table 3). Women who had ever been homeless, had a higher subjectively defined social status, and poorer mental health were associated with 4 or more visits, while visit frequency also varied by trial site (but was not subsequently entered into the final model) (table 3). Homelessness (OR=1.80, 95% CI=1.02 to 3.17) and subjective social status (OR=1.13, 95% CI=1.01 to 1.27) were the only two individual characteristics that remained independently associated with visit numbers.

For peer review only - http://bmjopen.bmj.com/site/about/guidelines.xhtml

For peer review only

Table 3 Baseline predictors of number of home visits from health visitor by six months postpartum for women in usual care arm

	3 or fewer visits (N=155) N	Median (IQR[a]) or %	4 or more visits (N=312) N	Median (IQR[a]) or %	Overall N=467) Median (IQR[a]) or N (%)	Univariate assoc.[g]
**Age in years**		17.9 (17.1 – 18.7)		17.8 (16.9 – 18.9)	17.8 (16.9 – 18.8)	0.721
**Ethnic background**						0.070
White	130	83.9	276	88.5	406 (86.9)	
Mixed	5	3.2	18	5.9	23 (4.9)	
Asian	3	1.9	4	1.3	7 (1.5)	
Black	15	9.7	13	4.2	28 (6.0)	
Other	2	1.3	1	0.3	3 (0.6)	
**Relationship status**						0.433
Married	4	2.6	3	1.0	7 (1.5)	
Separated	13	8.4	34	10.9	47 (10.1)	
Closely inv. / boyfriend	120	77.4	244	78.2	364 (77.9)	
Just friends	18	11.6	31	9.9	49 (10.5)	
**Live with father of baby**						0.512
Yes	42	27.1	71	22.8	113 (24.2)	
No	108	69.7	212	67.9	320 (68.5)	
Not answered	5	3.2	29	9.3	34 (7.3)	
**Subjective social status:**						
**Family**	155	5.8 (5.0 – 7.0)	309	5.8 (5.0 – 7.0)	5.8 (5.0 – 7.0)	0.896
**Personal**	154	6.8 (5.0 – 8.0)	311	7.1 (6.0 – 8.0)	6.7 (6.0 – 8.0)	**0.007[g]**
**NEET[b]**:	138		266			0.210
Yes	45	32.6	105	39.5	150 (37.1)	
No	93	67.4	161	60.5	254 (62.9)	
**Receive any benefits**	154		311			0.776
Yes	48	31.0	101	32.4	149 (31.9)	
No	106	68.4	210	67.3	316 (67.7)	
Not answered	1	0.6	1	0.3	2 (0.4)	
**Ever been homeless**						**0.023[g]**
Yes	19	12.3	65	20.8	84 (18.0)	
No	136	87.9	247	79.2	383 (82.0)	
**Deprivation (IMDS)[c]**	154	40.4 (24.8 – 54.3)	308	38.0 (24.8 – 51.4)	38.8 (24.8 – 51.7)	0.175
**Health utility**						0.374
Perfect health	104	67.1	195	62.5	299 (64.0)	
Less than perfect health	51	32.9	115	36.9	166 (35.5)	
Not answered	0	0.0	2	0.6	2 (0.4)	
**Self-rated health**						0.227
Excellent	24	15.5	58	18.6	82 (17.6)	
Good	113	72.9	200	64.1	313 (67.0)	
Fair	17	11.0	48	15.4	65 (13.9)	
Poor	1	0.6	6	1.9	7 (1.5)	
**Limiting chronic illness**:						0.144
Yes	24	15.5	66	21.2	90 (19.3)	
No	131	84.5	246	78.8	377 (80.7)	

For peer review only

For peer review only - http://bmjopen.bmj.com/site/about/guidelines.xhtml

**Self-efficacy**[d]	151	29.7 (27.0 – 32.5)	308	29.9 (28.0 – 32.0)	29.8 (27.0 – 32.0)	0.604
**Adaptive functioning:** [e]						
**Difficulty in at least one basic skill**						0.674
Yes	36	23.2	78	25.0	114 (24.4)	
No	119	76.8	234	75.0	353 (75.6)	
**3 or fewer key life skills**						0.822
Yes	39	25.2	81	26.0	120 (25.7)	
No	116	74.8	229	73.4	345 (73.9)	
Missing	0	0.0	2	0.6	2 (0.4)	
**At least one burden**						0.080
Yes	55	35.5	87	27.9	142 (30.4)	
No	98	63.2	224	71.8	322 (69.0)	
Missing	2	1.3	1	0.3	3 (0.6)	
**Alcohol / drug use** [f]	147	1.2 (0.0 – 2.0)	296	1.3 (0.0 – 2.0)	1.3 (0.0 – 2.0)	0.212
**Antisocial behaviour**	154	2.0 (1.0 – 3.0)	310	2.3 (1.0 – 4.0)	2.2 (1.0 – 3.0)	0.088
**Social support**	155	85.7 (77.0 – 98.7)	310	85.8 (79.0 – 98.7)	85.8 (77.6 – 98.7)	0.491
**Relationship quality**	130	28.5 (26.0 – 32.0)	255	28.2 (26.0 – 32.0)	28.3 (26.0 – 32.0)	0.433
**Family resources**	150	13.5 (11.0 – 16.0)	296	13.5 (11.0 – 16.0)	13.5 (11.0 – 16.0)	0.884
**Psychological distress / Mental health**	155	20.3 (15.0 – 25.0)	311	21.8 (17.0 – 26.0)	21.3 (16.0 – 26.0)	0.025
**Trial site**						0.003[h]
1	1	0.6	10	3.2	11 (2.4)	
2	5	3.2	8	2.6	13 (2.8)	
3	14	9.0	15	4.8	29 (6.2)	
4	2	1.3	7	2.2	9 (1.9)	
5	8	5.2	10	3.2	18 (3.9)	
6	6	3.9	7	2.2	13 (2.8)	
7	7	4.5	7	2.2	14 (3.0)	
8	12	7.7	19	6.1	31 (6.6)	
9	13	8.4	26	8.3	39 (8.4)	
10	5	3.2	17	5.4	22 (4.7)	
11	7	4.5	30	9.6	37 (7.9)	
12	17	11.0	16	5.1	33 (7.1)	
13	7	4.5	35	11.2	42 (9.0)	
14	5	3.2	3	1.0	8 (1.7)	
15	11	7.1	26	8.3	37 (7.9)	
16	19	12.3	19	6.1	38 (8.1)	
17	8	5.2	30	9.6	38 (8.1)	
18	8	5.2	27	8.7	35 (7.5)	

a Interquartile range; b Definition of NEET: Not in education employment or training (applicable only to those whose academic age is >16 at baseline interview); c Higher IMD score indicated more deprivation; d Higher score indicates higher level of self-efficacy; e Higher score indicates better management of day-to-day lives and routines (for each of the three sub-scales); f CRAFFT screening test[11] for substance related risks and problems in adolescents; g italics indicate variable included in logistic regression, bold indicates variable remained significantly associated with number of visits in logistic model; h not modelled in regression analysis due to high number of levels ; i The three original scale items comprised having to care for someone with long-term illness or alcohol / drug problem, feeling that they had in/sufficient privacy, living with people who respondents wished were not around

Other services

For peer review only

For peer review only - http://bmjopen.bmj.com/site/about/guidelines.xhtml

Participants accessed a wide range of services encompassing healthcare (table 2), housing and financial support (table 4), education, childcare and other support services including social care (table 5). A small proportion of respondents reported accessing support for housing outside of their friends and family, mostly from the local authority (table 4). The small difference in reported rates between study arms would appear to have been in part attributable to additional assistance from the FNP family nurse. Most participants reported being in receipt of additional publicly funded financial support. For most participants this included income support, housing benefit and council tax reductions with similar rates between study arms reported. Smaller proportions of participants reported other forms of financial assistance related to employment, education or personal health (eg, Jobseekers allowance). The largest difference in reported rates between study arms was for those who received regular financial support from parents: 8.9% (FNP), 15.4% (UC).

Table 4 Participants (%) reporting housing and financial support by follow-up point (months)

	6		12		18		24	
	FNP n=511	*UC n=470*	*FNP n=514*	*UC n=483*	*FNP n=501*	*UC n=466*	*FNP n=595*	*UC n=559*
i) Source of housing support								
Anyone outside of friends or family	18.0	14.9	12.1	9.9	9.2	8.4	12.1	9.7
Local authority housing department	7.0	6.6	5.1	5.6	4.6	4.7	6.2	5.9
Family Nurse	4.1	-	3.1	-	2.2	-	5.4	-
ii) Source of financial support								
State benefits or payments	-		-		-		86.9	88.4
Income support	-		-		-		62.0	63.3
Jobseekers allowance	-		-		-		8.6	8.9
Housing benefit	-		-		-		64.2	68.5
Council tax reduction	-		-		-		62.9	63.3
Disability living allowance	-		-		-		2.5	5.4
Incapacity benefit	-		-		-		0.7	1.6
Child Support Agency[1]	-		-		-		12.8	11.6
Regular support from parents	-		-		-		8.9	15.4
Education grants	-		-		-		5.5	5.9

1 Directly or via partner

Most women seeking contraception obtained it from their general practice, and to a lesser extent from a family planning clinic. There were some small differences between study arms by time point (eg at 18 months 46.1% of women in the UC arm accessed contraception from their GP, while 38.5% in the FNP did) but overall use of this service was similar. The proportion of women accessing any education gradually increased across the duration of the trial. By 24 months about a fifth of women were in school, college or training (FNP: 22.5%, UC: 18.1%). This was mostly in mainstream education, although there were a small number of women in both trial arms accessing support in more specialised units (eg, learning support unit). A similar pattern of increasing support for childcare was observed over time with approximately a quarter of women reporting some form of childcare support used at 24 months. Support was received from a variety of sources and there appeared to be a similar pattern of usage between study arms.

For peer review only - http://bmjopen.bmj.com/site/about/guidelines.xhtml

Table 5 Participants (%) reporting access to education, childcare and other support services by follow-up point (months)

	6		12		18		24	
	FNP n=511	*UC n=470*	*FNP n=514*	*UC n=483*	*FNP n=501*	*UC n=466*	*FNP n=595*	*UC n=559*
i) Education attended								
Any school, college or training	14.5	16.4	20.4	19.0	22.4	20.6	22.5	18.1
Mainstream school or college	11.3	13.7	15.0	15.6	19.5	18.7	16.6	12.7
Learning support unit	0.6	0.2	0.6	0.6	0.2	0	0.7	0.7
Pupil referral unit	0	0.2	0	0	0	0	0	0.2
Teenage mums support unit	0.8	1.7	0.6	0.6	0.4	0.6	0.7	1.5
ii) Childcare accessed								
Any childcare	7.0	7.0	16.1	13.3	25.5	21.5	26.9	24.3
Crèche at school or college	4.1	4.5	8.8	6.6	4.8	3.6	12.1	12.3
Day nursery at children's centre	0.8	0.6	0	0	3.6	2.4	5.5	4.3
Child-minder	1.8	1.1	2.1	1.2	3.2	2.4	3.2	3.0
Other forms of childcare	0.8	0.6	2.1	2.9	8.0	6.9	6.7	6.1
iii) Other support services								
Connexions	31.1	26.8	23.5	23.2	16.8	17.0	*	*
School nurse	1.4	1.5	0.8	0.4	0	0.9	0.5	0.9
Young People's Centre	4.9	7.0	2.7	3.9	1.8	1.9	1.8	1.6
Family Information Centre	2.0	2.3	1.2	1.5	2.2	3.0	1.3	1.4
Children's Centre	36.6	36.6	25.8	35.6	28.3	30.0	34.6	26.7
Child development centre	0.6	0.6	0.4	1.7	0.8	1.5	1.0	2.5
Crèche/ day nursery	10.8	10.8	15.4	14.7	8.4	6.0	17.6	16.6
Toddler group	7.8	7.9	12.5	11.0	16.2	15.2	19.2	21.5
Leaving care service	1.4	0.4	1.8	1.0	1.4	0.6	2.0	0.9
Fostering service	0.6	0.2	0.4	0.4	0	0.6	0.3	0.4
Youth offending team	0.8	0.9	0.2	0.2	0.4	0	0.3	0
Social worker	10.6	10.0	7.4	7.5	8.2	6.2	13.1	9.7
Alcohol / drug support	0.6	0	0.2	0.2	0	0.4	0.3	0.5

1 Some respondents indicated they were in school, college or training but provided no further information
** Not collected as service reconfigured*

For peer review only

Various other services were accessed, the most frequent being Connexions and Children's Centres. The former was used with decreasing frequency over time (consistent with the aging profile of the sample), while the latter showed a more variable pattern of access across each time point and on occasions quite different rates of access between trial arms. At six months one in ten mothers in both trial arms reported contact with a social worker, a rate that varied over time to 24 months at which point there was only a small difference between groups (FNP: 13.1%, UC: 9.7%).

DISCUSSION

To understand the service context within which FNP was trialled we mapped the range of services available. The multiplicity of services often within the same area and their varying labels often concealed similarities and differences between services. We established the usage of key services by trial participants across service domains. We particularly focused on those most directly relevant to the intervention (eg, health visiting) although included many other services. With mostly only small differences in usage between trial arms perhaps what is most important is the wide range of services being accessed. Although the previous US

For peer review only - http://bmjopen.bmj.com/site/about/guidelines.xhtml

trials have not further reported on broader services, the contexts were likely to be very different from the English trial setting.

FNP aims to impact upon a range of maternal and child outcomes. Therefore our selection of relevant services was necessarily broad and informed by the intervention's theory of change, which includes promoting access to services. However, previously reported attempts to map services have been challenging even when restricted to a single organisation.[12] To cope with such complexity researchers have sought to distinguish between specialist and generic services, including through a multi-staged approach as used here.[13] It has been consistently reported that information provision is time consuming for professionals (or other key informants) in such exercises, as we also found.[14] Individual informants may be unfamiliar with all relevant services even within their professional area, hence the coordinated approach to data gathering from multiple informants we used. Feedback from FNP staff in our process evaluation focus groups highlighted a similar challenge when acquiring knowledge about local services, essential for then linking up clients to relevant support.[10] Some core services such as mainstream education were not always reported and illustrates the need to clearly define the scope of the information request to informants, especially the boundaries within which they are being asked to respond. On this last point we would also clarify that many services however resourced and whether universal in availability or not, may impact upon the health and wellbeing of mother and child. We have measured for trial participants services actually used. The extent to which mothers can practically access currently unused or underused services effectively represents a key potential for future benefit if addressable barriers to accessed can be removed.

Our experience from this study will encourage us to further develop an approach to better understanding usual care in complex service settings. Our approached spanned an elicitation phase whereby we started by plotting a map of services and then a consolidation phase where we largely sought to confirm the contours on the map. Accordingly we took an exploratory approach for the former and a largely confirmatory approach for the latter. How either is actually done may depend on study setting and resource. The spreadsheets worked well in that they were portable and could be transferred easily to informants for completion once we had piloted them. However, an in-person semi-structured approach could have worked as well, but may have been more resource intensive. The complexity and number of services identified would have been unfeasible to include in their entirety in the trial's participant follow-up survey, but that may be important in some other studies. For example, if it was considered that sites clearly varied in provision of key services, gaining high quality information about such site characteristics could inform more informative analysis such as multi-level modelling. Finally, we initially explored the nature of available services with professionals, and only then asked mothers about services actually used via a mostly structured list of options. An exploratory exercise with mothers may well have shed light on other potential relevant services.

In effectiveness trials existing services could respond by augmenting support to those in the control arm. Such performance bias limits generalizability especially if that support was very different from usual care and approaching the level of support provided by the new intervention. Our findings do not indicate this in general and specifically for community midwifery and health visiting, the two most closely aligned universal services. However determining only the number of contacts may mask enhanced support provided in the form of longer contacts, or contacts from specialist practitioners. Community midwives visits were equivalent between trials arms and the difference in contacts with health visitors was attributable to clinic rather than home visits and therefore unlikely to be substantial. There was some indication that women in the usual care arm with some additional objective need identified at baseline, such as experience of homelessness, received more home visits. However, providing enhanced care to clients most in need would be usual practice. Evidence that this occurred in a trial context is not in itself a threat to external validity. The large caseloads managed by health visitors emphasises the lack of opportunity to provide significant additional support to mothers allocated to usual care.[10]

Our trial found fewer short-term benefits than previous US trials despite FNP being well implemented.[1-3, 8] The population we studied differed from that in the US, for example by

For peer review only - http://bmjopen.bmj.com/site/about/guidelines.xhtml

being fundamentally identified by maternal age and this may have contributed to some differences in impact detected. The upper age limit for women in the US trials was greater in each case than in England, and they also could have been enrolled at a later stage of gestation, for example, before delivery in Denver. In the three US trials the intervention had been provided by a total of 5 (Elmira), 12 (Memphis) and 10 (Denver) nurses in single areas with study samples of 400, 1138 and 735 women respectively. In our trial 131 nurses delivered the intervention across 18 local sites. The English service context would have been very different. Some additional standardised support in the form of developmental screening and referral, and free travel to appointments was provided to women in the control arm of each US trial. However, the broad and layered range of services identified in our study would not have been available. The broader adverse social context present in the first US trial and from which much longer-term evidence has been derived has limited direct comparison. For example, at the inception of the first US trial, Elmira was ranked bottom of all 380 US metropolitan statistical areas in terms of economic conditions. That is not to say that women in our trial were free of disadvantage or had services that fully met their needs. However, substantial differences across trial settings and the substantial duration between the trials are likely to have varied the potential for beneficial impact.

Service provision may change over time and any single mapping exercise will miss this real-world dynamic. We conducted telephone interviews with five FN supervisors towards the end of the trial. These explored whether there had been any key changes to local service provision. Recent major change was mostly not identified as occurring although the reduction in Connexions services was flagged up. Quantification of service use should be open to the capture of newer services. Additionally, with superficial service names not always reflecting well actual support provided it is important to look beyond service labels. Finally, high-level service descriptions do not always represent the often complex multi-professional interactions which necessarily facilitate service delivery. This emphasises the need for adequate qualitative description and interpretation of services.

Loss to follow-up at assessment points may introduce bias into the descriptive analysis. We have previously reported on group differences in attrition apparent at 24 months follow-up however such, differences were small.[10] A second consideration is the level of detail available for health visitor and midwifery contacts (eg, visit duration). It is reasonable to assume that given capacity and opportunity, women in the UC arm visited by health professionals would have received greater attention than other clients perceived as less in need. This is consistent with their professional role and reflective of contemporary best usual practice.[15] It is also possible that women in the FNP arm received relatively less attention than non-FNP clients if they were seen to be receiving enhanced support. Nevertheless, the total number of home and clinic visits received in both trial arms was small compared to that provided by FNP nurses. Future process evaluations should model the impact upon existing services of such service innovation to both avoid unintended consequences (eg, service displacement) and maximise synergy across services.

Moore and colleagues recommend primarily qualitative methods for capturing unanticipated or complex intervention pathways, which in this instance we take to be impact upon co-existing services.[16] They also emphasised the need to capture the mechanisms using logic models including where these reflected broader context. The extent to which an intervention's impact could actually induce harm either at the individual level or within a system can further be reflected by use of a dark logic model.[17] Bonell and colleagues recommend approaches to developing such a logic model, for example, by hypothesising how the agency of key stakeholders may interact with social structures to produce unintended consequences. Reflection in such model building could be informed by the use of mid-range sociological or psychological theory. This could also be combined with exploratory qualitative work with local stakeholders (eg service managers or practitioners) well placed to observe both intended and unintended intervention impacts. This is also consistent with approaches which recognise the implementation of public health interventions occurring within complex adaptive social systems, such as May's Normalisation Process Theory.[18] NPT identifies implementation as occurring in a dynamic, non-linear and emergent fashion. This offers a broader theoretical context within which to explore not only how one intervention becomes adapted to its

For peer review only - http://bmjopen.bmj.com/site/about/guidelines.xhtml

environment and may vary but also how that social context and usual services may also become adapted too.

The effectiveness of a public health intervention can only be adequately evaluated with a sound understanding of the service context within which it operates and which may also form the trial comparator.[19] Describing and quantifying the nature of usually available services can be challenging especially when services arise from a number of sectors, may evolve over the period of study and vary across study sites. In mapping the pattern of support potentially available to participants in our trial we have gained a critical understanding of the context within which and against which FNP should be considered. In quantifying maternal reported service usage we have provided key insights into how our main trial results should therefore be interpreted. While challenging, we remain convinced of the need to develop this area of research when evaluating public health interventions. Indeed, in their feedback survey respondents reported the usefulness of the exercise in gaining greater insights about local services, some sharing the generated service summaries with their teams.

For peer review only

For peer review only - http://bmjopen.bmj.com/site/about/guidelines.xhtml

ACKNOWLEDGEMENTS
The authors gratefully acknowledge the contribution of Dr Lesley Wye, University of Bristol and Dr Marie-Jet Bekkers who contributed to some of the planning and implementation of the reported study, and to all other members of the broader Building Blocks research team who supported overall trial delivery. We are grateful to each local site informant who contributed to data collection in either of the two rounds of service mapping and especially to the three local coordinators who initially piloted our mapping tool. We are also grateful to all women who participated in the trial and for their time in completing outcome and resource assessments.

COMPETING INTERESTS
Prior to working on the Building Blocks trial, Sue Channon facilitated two workshops on Motivational Interviewing for supervisors within the FNP. The authors declare no competing interests.

CONTRIBUTORS
MR, KH and JS conceived the study and all authors contributed to the development of its protocol. MR wrote the first draft with further contributions from all authors. RP, JS and GM were involved in data collection and management. RP was responsible for developing the survey of local stakeholders and GM was responsible for managing data collected from trial participants used in the analysis. RP, RCJ, GM and MR were involved in analysis and developing summary tables for publication. SC and JS were responsible for the management of this package of work within the trial overall. MR was responsible for obtaining study funding. All authors contributed to data interpretation, reviewed successive drafts and approved the final version of the manuscript.

ETHICS
The trial was approved by the Wales NHS Research Ethics Committee (09/MRE09/08) and received governance approval from all participating NHS sites. All women provided written informed consent.

PARTICIPANT CONSENT
All participants provided written informed consent to take part in the study.

FUNDING
The Centre for Trials Research, Cardiff University is funded by the Welsh Government through Health and Care Research Wales and the authors gratefully acknowledge the Centre’s contribution to study implementation. This is an independent report commissioned, sponsored and funded by the Policy Research Programme in the Department of Health (reference no. 006/0060). The views expressed are not necessarily those of the Department of Health.

DATA SHARING
The datasets generated and analysed during the current study are not publicly available as contributors / participants may be identifiable and are also subject to sponsor approval, but may be available from the corresponding author on reasonable request.

For peer review only

For peer review only - http://bmjopen.bmj.com/site/about/guidelines.xhtml

References

1. Olds DL, Henderson CR, Jr., Chamberlin R, Tatelbaum R. Preventing child abuse and neglect: a randomized trial of nurse home visitation. Pediatrics 1986; 78 (1): 65-78.
2. Kitzman H, Olds DL, Henderson CR, Jr., et al. Effect of Prenatal and Infancy Home Visitation by Nurses On Pregnacy Outcomes, Childhood Injuries, and Repeated Childbearing: A Randomised Controlled Trial. JAMA 1997; 278 (8): 644-52.
3. Olds DL, Robinson J, O'Brien R, et al. Home Visiting by Paraprofessionals and by Nurses: A Randomized, Controlled Trial. Pediatrics 2002; 110 (3): 486-96.
4. Barnes J, Ball M, Meadows P et al. Nurse-Family Partnership Programme: First Year Pilot Sites Implementation in England - Pregnancy and the Post-partum Period. London: Birkbeck, University of London; 2008.
5. Barnes J, Ball M, Meadows P et al. Nurse-Family Partnership Programme: Second Year Pilot Sites Implementation in England - The Infancy Period. London: Birkbeck, University of London; 2009.
6. Barnes J, Ball M, Meadows P, et al. The Family-Nurse Partnership Programme in England: Wave 1 implementation in toddlerhood & a comparison between Waves 1 and 2a of implementation in pregnancy and infancy. London: Institute for the Study of Children, Families and Social Issues: Birkbeck, University of London, 2011.
7. Owen-Jones E, Bekkers M-J, Butler CC, et al. The effectiveness and cost-effectiveness of the Family Nurse Partnership home visiting programme for first time teenage mothers in England: a protocol for the Building Blocks randomised controlled trial. BMC Pediatrics 2013; 13 (1): 114.
8. Robling M, Bekkers M-J, Bell K, et al. Effectiveness of a nurse-led intensive home-visitation programme for first-time teenage mothers (Building Blocks): a pragmatic randomised controlled trial. Lancet 2015; published online Oct 14. http://dx.doi.org/10.1016/S0140-6736(15)00392-X.
9. NVivo qualitative data analysis Software; QSR International Pty Ltd. Version 8, 2008
10. Robling MR Bekkers M-J, Bell K et al. Evaluating the Family Nurse Partnership Programme in England: A Randomised Controlled Trial. Final report to The Department of Health. 2015. (http://bit.ly/buildingblockstrial).
11. Knight JR, Sherritt L, Shrier LA et al Validity of the CRAFFT substance abuse screening test among adolescent clinic patients. Arch Pediatr Adolesc Med 2002, 156(6):607–614.[CRAFFT].
12. Hotopf M, Rayner L, Valsraj K. Improving 'Whole Person Care' A report on mental health related services offered by King's Health Partners' acute trusts: King's Health Partners, 2012.
13. Challis D, Darton R, Donnelly M et al. Mapping and evaluation of care management arrangements for older people and those with menatal health problems in an integrated health and social services system: a comparative study: The Personal Social Services Research Unit, 1999.
14. Health Do. Children's services mapping. A complete picture of children's service provision: Department of Health. Department for Children Schools and Families, 2010.
15. NICE. Pregnancy and complex social factors: A model for service provision for pregnant women with complex social factors. London, 2010.
16. Moore GF, Audrey S, Barker M, Bond L, Bonell C, Hardeman W, Moore L, O'Cathain A, Tinati T, Wight D, Baird J. Process evaluation of complex interventions: Medical Research Council guidance. BMJ 2015;350:h1258 doi:10.1136/bmj.h1258
17. Bonell C, Jamal F, Melendez-Torres GJ, Cummins S. 'Dark logic': theorising the harmful consequences of public health interventions. Epidemiol Community Health 2015;69:95–98.
18. May CR, Johnson M, Finch T. Implementation, context and complexity. Implementation Science (2016) 11:141
19. Smelt AFH, van der Weele GM, Blom JW et al. How usual is usual care in pragmatic intervention studies in primary care? An overview of recent trials. British Journal of General Practice 2010 e305.

For peer review only - http://bmjopen.bmj.com/site/about/guidelines.xhtml

Appendix 1

Example of service mapping content / user instructions from 'Education' worksheet

Please describe services available for teenage, first-time mothers in your area	**Name of service (If applicable)**	**Description of service / Care**	**Are there any limits on the availability of this service? E.g., Number of women that can be offered the service**
Is there a Mother and Baby hostel in your area?	Mother and baby hostels	Dedicated hostels for homeless women who are pregnant or have a new baby. Additional support both from trained staff	Some will take young women in the early stages of pregnancy (up to 6 months approx). Some will not house pregnant women, or young babies
What temporary accommodation can be offered?	Hostels	Temporary accommodation with varying degrees of support, usually containing some shared facilities	Most require assessment of eligibility and suitability. Not usually suitable for those under 18, or youths with offending behaviour.
Temporary accommodation (for the temporary homeless)?	Emergency hostels and night shelters (or bed and breakfasts if nothing else is available - usu max of 6 weeks)	Offer somewhere to sleep, food, warmth and hygiene. Residents are normally asked to pay a small additional contribution for their meals.	Usually a direct access/first-come-first served basis. Night shelters are usually free. Most hostels charge.
Charity involvement?	LIFE Housing	Provides a support service (General Support, Individual Support Plans and the LIFE Skills Programme) and community outreach schemes	(Not specified)
Foyers	Foyers	Integration of accommodation and support services: training in basic/independent living skills, inc. ongoing support when the young person has left the Foyer	Most foyers have a waiting list. Some foyers only accept referrals from local councils
Women's refuges	Women's refuges	A refuge is a safe house where women and children who are experiencing domestic violence can stay free from abuse	Refuges are highly unlikely to accept women from their immediate local area. Some are for women w/ part' ethnic/cultural backgrounds
Housing Associations / RSLs (Registered Social Landlords)	Housing associations / RSLs (Registered Social Landlords)	Provide homes for people on low incomes. Some housing associations specialise in accommodation for particular groups of people, such as younger people	Long waiting list. Chance of place/waiting time depends on personal circumstances (e.g. children), and other factors
Supported lodgings schemes	Supported lodgings	Individuals in the community offer a room in their home with varying degrees of support. A safe and supportive environment for young people	Suitable for youths: leaving care, deemed vulnerable and in need, requiring temp acc, or with no statutory entitlement to housing

For peer review only

Supported housing	Self-contained, cluster and shared arrangements with varying degrees of support	Supported housing will usually provide housing-related support to help a young person prepare for independent living	Many schemes accessible via social services' leaving care arrangements, and via housing departments for young homeless people
Mainstream housing	Independent accommodation	Independent accommodation. Housing & social services may have arrangements w/ private landlords to provide accommodation for vulnerable youths	Housing benefit restrictions apply to under 25s living in the private sector and applicants are limited to the single-room rent housing benefit level
Nightstop schemes	Emergency accommodation (Depaul Nightstop UK)	Nightstop schemes provide emergency accommodation for young homeless people aged 16–25 in the homes of a network of volunteer hosts	Referrals to service after risk assessments by a recognised agency; such as social services, Connexions, police, housing depts
Supported housing	Supported housing schemes	Housing schemes offering accommodation linked with on-site or outreach support from dedicated staff (practical and emotional help)	Schemes vary in size
Custodial institution	Secure Children's Homes	Concentrate on physical, emotional, behavioural needs, & aim to give youths individually tailored support to resolve the issues that led them to commit an offence	These are relatively small institutions, with between 6 and 40 beds and a high staff to young person ratio
Floating support	Floating support services	To sustain a tenancy through the dev' of independent living skills. Provides general, non-specialist support with daily living skills, practical tasks or emotional support which promotes or maintains a person's ability to live in their own home	Reviews of the progress and support plan ensure that the services adjust appropriately to changing needs. An exit strategy determines when support is withdrawn. If a person needs support later, it can return to them

For peer review only

Building Blocks Service Mapping Report

STROBE Statement—checklist of items that should be included in reports of observational[1] studies

	Item No	Recommendation	Page
**Title and abstract**	1	(*a*) Indicate the study's design with a commonly used term in the title or the abstract	1
		(*b*) Provide in the abstract an informative and balanced summary of what was done and what was found	2
**Introduction**			
Background/rationale	2	Explain the scientific background and rationale for the investigation being reported	3
Objectives	3	State specific objectives, including any prespecified hypotheses	3
**Methods**			
Study design	4	Present key elements of study design early in the paper	3, 4
Setting	5	Describe the setting, locations, and relevant dates, including periods of recruitment, exposure, follow-up, and data collection	4, 5, 6, 9
Participants	6	(*a*) *Cohort study*—Give the eligibility criteria, and the sources and methods of selection of participants. Describe methods of follow-up<br>*Case-control study*—Give the eligibility criteria, and the sources and methods of case ascertainment and control selection. Give the rationale for the choice of cases and controls<br>*Cross-sectional study*—Give the eligibility criteria, and the sources and methods of selection of participants	5
		(*b*) *Cohort study*—For matched studies, give matching criteria and number of exposed and unexposed<br>*Case-control study*—For matched studies, give matching criteria and the number of controls per case	NA
Variables	7	Clearly define all outcomes, exposures, predictors, potential confounders, and effect modifiers. Give diagnostic criteria, if applicable	10-14
Data sources/ measurement	8*	For each variable of interest, give sources of data and details of methods of assessment (measurement). Describe comparability of assessment methods if there is more than one group	*10-14*
Bias	9	Describe any efforts to address potential sources of bias	NA
Study size	10	Explain how the study size was arrived at	4
Quantitative variables	11	Explain how quantitative variables were handled in the analyses. If applicable, describe which groupings were chosen and why	5
Statistical methods	12	(*a*) Describe all statistical methods, including those used to control for confounding	5
		(*b*) Describe any methods used to examine subgroups and interactions	NA
		(*c*) Explain how missing data were addressed	9*
		(*d*) *Cohort study*—If applicable, explain how loss to follow-up was addressed<br>*Case-control study*—If applicable, explain how matching of cases and controls was addressed<br>*Cross-sectional study*—If applicable, describe analytical methods taking	NA

For peer review only

		account of sampling strategy	
		(*e*) Describe any sensitivity analyses	NA

**Results**			
Participants	13*	(a) Report numbers of individuals at each stage of study—eg numbers potentially eligible, examined for eligibility, confirmed eligible, included in the study, completing follow-up, and analysed	9*
		(b) Give reasons for non-participation at each stage	9*
		(c) Consider use of a flow diagram	9*
Descriptive data	14*	(a) Give characteristics of study participants (eg demographic, clinical, social) and information on exposures and potential confounders	9*
		(b) Indicate number of participants with missing data for each variable of interest	10-14
		(c) *Cohort study*—Summarise follow-up time (eg, average and total amount)	10-14
Outcome data	15*	*Cohort study*—Report numbers of outcome events or summary measures over time	*10-14*
		Case-control study—Report numbers in each exposure category, or summary measures of exposure	-
		Cross-sectional study—Report numbers of outcome events or summary measures	-
Main results	16	(*a*) Give unadjusted estimates and, if applicable, confounder-adjusted estimates and their precision (eg, 95% confidence interval). Make clear which confounders were adjusted for and why they were included	10-14
		(*b*) Report category boundaries when continuous variables were categorized	NA
		(*c*) If relevant, consider translating estimates of relative risk into absolute risk for a meaningful time period	NA
Other analyses	17	Report other analyses done—eg analyses of subgroups and interactions, and sensitivity analyses	NA
**Discussion**			
Key results	18	Summarise key results with reference to study objectives	14,15
Limitations	19	Discuss limitations of the study, taking into account sources of potential bias or imprecision. Discuss both direction and magnitude of any potential bias	15,16
Interpretation	20	Give a cautious overall interpretation of results considering objectives, limitations, multiplicity of analyses, results from similar studies, and other relevant evidence	14, 15, 17
Generalisability	21	Discuss the generalisability (external validity) of the study results	16
**Other information**			
Funding	22	Give the source of funding and the role of the funders for the present study and, if applicable, for the original study on which the present article is based	18

1 The paper reports an analysis from the process evaluation (including descriptive analysis of service usage data) from a cohort of women participating in a randomised control trial

** Citation to full main trial report included in text*

For peer review only - http://bmjopen.bmj.com/site/about/guidelines.xhtml
